# Source‐specific nitrate intake and incident dementia in the Danish Diet, Cancer and Health Study

**DOI:** 10.1002/alz.70995

**Published:** 2025-12-19

**Authors:** Catherine P. Bondonno, Pratik Pokharel, Dorit Wielandt Erichsen, Liezhou Zhong, Jörg Schullehner, Cecilie Kyrø, Kirsten Frederiksen, Peter Fjeldstad Hendriksen, Frederik Dalgaard, Lauren C. Blekkenhorst, Stephanie R. Rainey‐Smith, Samantha L. Gardener, Torben Sigsgaard, Ole Raaschou‐Nielsen, Anne Tjønneland, Jonathan M. Hodgson, Christina C. Dahm, Anja Olsen, Nicola P. Bondonno

**Affiliations:** ^1^ School of Medical and Health Sciences Edith Cowan University Joondalup Western Australia Australia; ^2^ Medical School, Royal Perth Hospital The University of Western Australia Perth Western Australia Australia; ^3^ Diet, Cancer and Health Group The Danish Cancer Institute Copenhagen Denmark; ^4^ Department of Geochemistry Geological Survey of Denmark and Greenland Aarhus Denmark; ^5^ Department of Public Health Aarhus University Aarhus Denmark; ^6^ Department of Cardiology Herlev & Gentofte University Hospital Copenhagen Denmark; ^7^ Centre for Healthy Ageing, Health Futures Institute Murdoch University Murdoch Western Australia Australia; ^8^ Centre of Excellence for Alzheimer's Disease Research & Care, School of Medical and Health Sciences Edith Cowan University Joondalup Western Australia Australia; ^9^ Danish Big Data Centre for Environment and Health (BERTHA) Aarhus University Roskilde Denmark; ^10^ Department of Environmental Science Aarhus University Roskilde Denmark; ^11^ Department of Public Health University of Copenhagen Copenhagen Denmark

**Keywords:** cohort study, early‐onset dementia, incident dementia, nitrate, nitrite

## Abstract

**INTRODUCTION:**

Dietary nitrate, through conversion to nitric oxide, which supports vascular and nervous system function, may lower dementia risk but may also form neurodegenerative *N*‐nitrosamines, depending on the nitrate source.

**METHODS:**

We investigated associations between source‐specific nitrate and nitrite intake and incident and early‐onset dementia (<65 years) in 54,804 dementia‐free participants from the Danish Diet, Cancer and Health Cohort Study over ∼27 years. Nitrate and nitrite intakes were derived from food frequency questionnaires and nitrate and nitrite databases.

**RESULTS:**

Higher plant‐sourced nitrate intake was non‐linearly associated with lower rates of incident dementia (fifth vs first quintile hazard ratio 95% confidence interval: 0.90 [0.83, 0.98]), while increased risk was seen for higher intakes of animal‐sourced, additive‐permitted meat‐sourced, and tap water‐sourced nitrate. Similar associations were seen for source‐specific nitrite intake and were more pronounced for early‐onset dementia. No clear effect modification was observed.

**DISCUSSION:**

These findings highlight the importance of nitrate source in dementia risk and warrant further investigation.

**Highlights:**

- Plant nitrate is associated with a lower risk of incident and early‐onset dementia.
- Animal and tap water nitrate are associated with an increased risk of dementia.
- Encouraging consumption of plant‐based nitrate sources may lower risk of dementia.

## 1 BACKGROUND

The impact of dietary nitrate on human health remains a topic of debate. Dietary nitrate comes from plants, animal‐derived foods (where it is both naturally occurring and a permitted food additive), and water, with the majority of intake coming from green leafy vegetables and certain root vegetables.^1^ Nitrate exerts its beneficial effects through its derivative, nitric oxide (NO), a key signaling molecule that regulates both the vascular and nervous systems.^2^, ^3^ Clinical studies demonstrate that nitrate positively influences NO‐mediated processes such as lowering blood pressure and improving endothelial function.^4^, ^5^ Observational evidence consistently links diets rich in vegetable‐sourced nitrate with a lower risk of cardiovascular disease (CVD).^6^, ^7^, ^8^, ^9^ Although nitrate's vascular actions are well established, its positive influence on NO may also extend to other health conditions such as dementia. Beyond its vascular role, NO is also a neuronal signaling molecule playing a key role in cerebrovascular protection and neuronal function.^10^, ^11^ Furthermore, the overlap between risk factors for CVD and dementia^12^ highlights the importance of vascular health in cognitive aging, suggesting that nitrate could plausibly contribute to dementia prevention through neuroprotective effects and vascular regulation.

Contrary to the known and potential beneficial effects of nitrate intake, nitrate and its metabolite nitrite have the potential to form *N*‐nitrosamines, which are linked with cancer,^13^, ^14^ Preclinical studies indicate that *N*‐nitrosamines also have neurodegenerative effects.^15^ The contrast in potential health effects has led to the hypothesis that the positive or negative health effects of dietary nitrate are dependent on the source of intake.^6^, ^16^ Plants contain compounds such as polyphenols, folate, and vitamins C and E, which inhibit the formation of *N*‐nitrosamines,^6^ while meat contains heme and amines, which may promote the formation of *N*‐nitrosamines.^17^ Recent observational cohort studies support this hypothesis with source‐dependent differential associations observed for all‐cause mortality and CVD.^9^, ^18^ Furthermore, source‐dependent differential associations have been observed for dementia mortality and cognitive function in small cohorts.^19^, ^20^ Clinical and observational studies have shown that dietary interventions such as the Mediterranean Diet, Dietary Approaches to Stop Hypertension (DASH), the Mediterranean‐DASH Diet Intervention for Neurodegenerative Delay (MIND), and multidomain approaches like the FINGER trials, which incorporate healthy diet adherence, are associated with a reduction in dementia risk.^21^, ^22^, ^23^, ^24^ Notably, these diets align with the nitrate source hypothesis, emphasizing high consumption of vegetables – particularly green leafy vegetables, the richest sources of nitrate – along with lower intakes of processed meat, where nitrate is an additive.

In view of the potential source‐dependent effects of nitrate on dementia risk, the primary aim of this study was to investigate the association between habitual intake of nitrate and nitrite from different sources and incident dementia in the Danish Diet, Cancer and Health Cohort Study. Sources include all plants (including vegetables), animal‐based foods where nitrate/nitrite naturally occur, meat products where nitrate/nitrite are regulated additives, and drinking water (nitrate only). Given the recent observation that the magnitude of associations between risk factors such as diet and dementia may be larger for early‐onset dementia,^25^ a secondary aim of the current study was to investigate associations between source of nitrate/nitrite with early‐onset dementia. An additional aim was to determine whether associations differed in the presence of factors hypothesized to promote (smoking) or inhibit (concomitant intakes of polyphenols, folate, and vitamins C and E) the conversion of nitrate to harmful *N*‐nitrosamines.

## 2 MATERIALS AND METHODS

### 2.1 Study population

The Danish Diet, Cancer and Health Cohort Study recruited 57,053 males and females residing in the greater areas of Copenhagen and Aarhus, Denmark, from December 1993 through May 1997. Participants were between the ages of 50 and 64 years at recruitment. The following databases were cross‐linked to the cohort using unique IDs assigned to all inhabitants of Denmark: Civil Registration System,^26^ National Death Registry,^27^ Danish National Patient Register (DNPR),^28^ Danish National Prescription Registry,^29^ Register for Selected Chronic Diseases (RUKS),^30^ and Education Registry.^31^ As RUKS only contains complete data from January 1, 1995, the baseline for the current study was set to this date for all participants recruited before 1995. We excluded participants who died or immigrated before January 1, 1995 (*n* = 16), participants with prevalent dementia (*n* = 55) at baseline, defined as a validated diagnostic record of dementia in the DNPR (International Classification of Diseases, Revision 8 [ICD‐8]: 29009–29011, 29018, 29019, 29309, and 29319; ICD‐10: F00–F02, F039, and G30) or a record of dementia in RUKS (the algorithm is described in more detail in what follows in Section 2.3, “Study outcomes,” and elsewhere^30^) prior to baseline. Additionally, participants with missing exposure or covariate data (*n* = 1590) were excluded, leaving 54,804 participants for analysis in this study (Figure S1).

**RESEARCH IN CONTEXT**

1. **Systematic review**: Although dietary nitrate is known to promote vascular and neurological health via nitric oxide production, its potential conversion to neurotoxic *N*‐nitrosamines, particularly from animal or water sources, raises concerns regarding dementia risk. Prior studies seldom distinguished between nitrate sources, limiting understanding of differential effects on dementia.
2. **Interpretation**: Our findings suggest that a higher intake of plant‐sourced nitrate is associated with a lower risk of incident and early‐onset dementia, whereas nitrate from animal products, processed meats, and tap water is linked to increased risk. Associations were more pronounced for early‐onset dementia, and no significant modification was observed for sex, smoking, or dietary components.
3. **Future directions**: The current findings require replication in diverse populations, and mechanistic studies are warranted to clarify causal pathways and to determine whether dietary interventions or water quality improvements can mitigate dementia risk associated with non‐plant nitrate sources.

The study was approved by relevant scientific ethics committees and the Danish Data Protection Agency, and all participants gave written informed consent.

### 2.2 Source‐specific nitrate and nitrite intake

Prior to their first visit, participants completed a 192‐item validated food frequency questionnaire (FFQ).^32^, ^33^, ^34^ Participants reported the frequency within 12 possible categories of their consumption of different food and beverage items over the previous 12 months. These intake frequencies were adjusted through additional “global” questions specifying total intakes of major food groups. Recipes and portion sizes for each food and beverage item were used to estimate the daily intake (g/day) of each food and nutrient using the software FoodCalc.^35^ A detailed description of the calculation of nitrate and nitrite intake from this FFQ was reported previously.^36^ The food and beverage items were categorized into four primary groups for our analysis: foods originating from plant sources, foods derived from animal sources, tap water, and other sources (specifically alcoholic beverages and discretionary foods). A brief description follows.

#### 2.2.1 Nitrate and nitrite intake from plant sources

The assessment of nitrate and nitrite intake from plant sources, comprising all plant‐based foods including fruits, vegetables, whole grains, nuts, and plant oils, was conducted using a comprehensive plant‐source nitrate/nitrite database.^1^ To account for the impact of cooking on nitrate content, a conservative estimate of a 50% reduction in nitrate content in raw vegetables was applied to cooked or boiled vegetables, as prior research indicated that boiling could reduce their nitrate content by approximately half.^1^ Nitrate/nitrite intake was computed by multiplying the reported daily consumption quantity of each ingredient (g) by its corresponding median nitrate/nitrite value (mg/g). The total dietary nitrate intake from plant‐based foods was subsequently determined as the sum of the nitrate content across all individual plant‐sourced ingredients.

Given that vegetables constitute the primary dietary source of nitrate^1^, we also examined associations for vegetable‐derived nitrate specifically for clearer public health recommendations.

#### 2.2.2 Nitrate and nitrite intake from animal‐based sources

To estimate nitrate and nitrite intake from animal sources, which encompass red meat, poultry, processed meat, offal, dairy, eggs, fish, and other seafood, an animal‐source nitrate/nitrite database was used.^37^ The methodology for assigning nitrate and nitrite values followed the approach described for plant sources. Additionally, we distinguished between intrinsic nitrate/nitrite (naturally occurring) and nitrate/nitrite used as permitted additives in animal‐sourced foods. This distinction is critical for informing public health policies.

#### 2.2.3 Nitrate intake from drinking water

To assess nitrate levels in tap water, we used the Danish national drinking water quality monitoring database Jupiter.^38^ Spatially linking this database to the historical addresses of cohort participants, an estimate of the individual‐level nitrate consumption derived from tap water spanning the years 1978 to 2016 was obtained. Baseline tap water nitrate intake was calculated using tap water consumption from the FFQ and multiplied by the time‐weighted average of nitrate concentration at every address each cohort participant lived at in the 12 months prior to their enrollment in the study. In addition, water nitrate concentration was estimated as the cumulative average concentration of nitrate in drinking water supplied to the addresses where each participant had lived over the previous 15 years. Both public and private well users were included in these analyses as no difference in associations were found when private well users were excluded in our previous study.^39^ Nitrate from drinking water exposures included baseline tap water nitrate intake, baseline tap water nitrate concentration, and time‐varying tap water nitrate concentration.

### 2.3 Study outcomes

The primary outcome of the study was incident dementia recorded in RUKS. This register defines dementia as the first validated diagnostic record of a hospital contact with dementia diagnosis (ICD‐10 codes: F00, F01, F02, F03, G30, G31.0B, G31.8, G31.8E, and G31.9) recorded in the DNPR, or the collection of prescribed medication for dementia (Anatomical Therapeutic Chemical code: N06D), recorded in the DNPR. Further details regarding the algorithm used can be found elsewhere.^30^ The secondary outcome was early‐onset dementia, defined as diagnosis before 65 years of age.^40^

### 2.4 Covariates

At the time of enrollment in the Danish Diet, Cancer and Health Study, participants provided information on their age, sex, smoking status (current/former/never), smoking intensity history (represented as pack‐years: lifetime average number of cigarettes smoked multiplied by the number of years smoked divided by 20) and daily physical activity (represented as total daily metabolic equivalent of task [MET] score) in self‐administered questionnaires. The height and weight of participants were measured at baseline, from which body mass index (BMI) (kg/m^2^) was computed. Information on each participant's education level was obtained from the Education Registry. Educational levels were categorized as follows. “Short” denoted mandatory schooling, limited to 7 years for those born before January 1, 1958, and 9 years for those born after. “Medium” encompassed secondary and vocational education, corresponding to 10 to 12 years of schooling. “Higher” encompassed various levels of tertiary education, typically exceeding 12 years of education. Information on living situations (living with a partner/single) was obtained from the Civil Registration System. The FFQ was used to estimate alcohol intake (g/day), total polyphenol intake (mg/day; calculated using the Phenol‐Explorer database^41^), folate intake (µg/day), vitamin C intake (µg/day), and vitamin E intake (mg α tocopherol equivalents/day). Dietary polyphenol, folate, vitamin C, and vitamin E intakes were categorized into tertiles with the highest and lowest tertiles reflecting “high” and “low” intakes, respectively. Prevalent CVD was defined as a diagnosis of ischemic heart disease, ischemic stroke, peripheral heart disease, heart failure, or atrial fibrillation in the DNPR prior to baseline (see Table S1 for further information). Prevalent chronic kidney disease (CKD) and prevalent chronic obstructive pulmonary disease (COPD) were defined as a record of the respective disease in either the DNPR or the RUKS, while prevalent diabetes was defined as a record of either type 1 or type 2 diabetes in RUKS (see Table S1 for further information). Presence of hypertension was defined by the use of two or more antihypertensive medications within 180 days prior to baseline, which has a positive predictive value of 80.0% and a specificity of 94.7% to predict hypertension.^42^, ^43^

### 2.5 Statistical analysis

Baseline characteristics of cohort participants were summarized overall as well as according to quintiles of plant‐derived nitrate, animal‐derived nitrate, and water‐derived nitrate intakes. Correlations between exposure variables and major food groups have been described previously.^36^ In total, 54,804 participants were followed from January 1, 1995 (the official start date of the RUKS register) until date of dementia diagnosis, death, or the end of follow‐up (December 31, 2020), whichever came first. When early‐onset dementia was the outcome of interest, participants were censored when they turned 65 years. Cumulative incidence plots of dementia by quintiles of each nitrate exposure were generated using the Aalen–Johansen estimator, accounting for death as a competing risk. Continuous exposure variables were modeled both as restricted cubic splines (with the reference set at the median intake of the lowest quintile, corresponding to the 10th percentile) and as log_2_‐transformed variables within Cox proportional hazards models to account for potential non‐linear relationships with incident dementia. The hazard ratios (HRs) and 95% confidence intervals (CIs) derived from the spline models were plotted, with HRs on the *y*‐axis and exposure intakes (cut at 3 standard deviations from the mean for clarity) on the *x*‐axis. In the tables, HRs and 95% CIs for the median intake in each quintile (i.e., at the 30th, 50th, 70th, and 90th percentiles) are extracted from the spline model and also presented for the log_2_‐transformed variables, with the interpretation for the latter being per doubling of the exposure. Proportional hazards assumptions were tested using log‐log plots of the survival function versus time and assessed for parallel appearance, with no violation observed. The following modeling strategy was used; Model 1 included age and sex; Model 2 included age, sex, BMI, smoking status, smoking pack‐years, alcohol consumption, education level, physical activity level, and living situation; Model 3 adjusted for the covariates in Model 2 plus intakes of (1) red meat, processed meat, poultry, dairy, fish, sugar and confections, soft drinks, refined grains, coffee, and tea when plant‐sourced nitrate or nitrite was the exposure of interest; (2) whole grains, refined grains, vegetables, fruits, vegetable oils, sugar and confections, soft drinks, coffee, and tea when animal‐sourced nitrate or nitrite was the exposure of interest; and (3) whole grains, refined grains, red meat, processed meat, poultry, dairy, fish, vegetables, fruits, vegetable oils, sugar and confections, soft drinks, coffee, and tea when water‐sourced nitrate was the exposure of interest. Covariates were chosen a priori using prior knowledge of potential confounders of diet and dementia associations. All continuous covariates were modeled with restricted cubic splines. To examine potential effect modification, analyses were stratified by sex, smoking status (ever/never), vitamin C (low/high), vitamin E (low/high), folate (low/high), and polyphenol (low/high) with the exposures modeled as log_2_‐transformed variables. These analyses were intended to examine patterns and consistency across subgroups rather than to provide formal tests of statistical interaction. To assess if associations were stronger when the dietary assessment was more temporally proximate to the outcome, a sensitivity analysis was performed by restricting the follow‐up period to 10 years. In a secondary analysis, drinking water nitrate concentration was modeled (1) as baseline concentration adjusted for baseline tap water intake and (2) as a time‐varying exposure variable with a restricted cubic spline. For the latter, at each age (as age was the underlying timescale), the exposure was estimated as the cumulative average concentration of nitrate in drinking water supplied to the addresses where each participant had lived over the previous 15 years and end of follow‐up was set as December 31, 2016. Age‐stratified analyses were conducted using Cox proportional hazards models with age as the timescale, implementing left truncation at the lower boundary and right censoring at the upper boundary of each age stratum to avoid immortal time bias. Analyses were undertaken using R statistics (R Core Team, 2022) and SAS 9.4 (SAS Institute, Cary, NC, USA).

## 3 RESULTS

The 54,804 study participants had a median (interquartile range [IQR]) age of 56^52^, ^53^, ^54^, ^55^, ^56^, ^57^, ^58^, ^59^, ^60^ years at study entry and a median (IQR) follow‐up time of 24^20^, ^21^, ^22^, ^23^, ^24^, ^25^ years. Over a maximum follow‐up of ∼27 years, 4750 individuals were defined as having dementia, with 191 cases defined as early‐onset dementia and 18,504 participants died. Median (IQR) age of diagnosis was 77.0 (72.9 to 81.1) years for total dementia and 62.0 (59.1 to 63.7) for early‐onset dementia. The median (IQR) of nitrate intake from plant sources was 44^31^, ^32^, ^33^, ^34^, ^35^, ^36^, ^37^, ^38^, ^39^, ^40^, ^41^, ^42^, ^43^, ^44^, ^45^, ^46^, ^47^, ^48^, ^49^, ^50^, ^51^, ^52^, ^53^, ^54^, ^55^, ^56^, ^57^, ^58^, ^59^, ^60^ mg/day, from animal sources it was 5.8 (4.1 to 8.4) mg/day, and from tap water it was 0.8 (0.2, 1.8) mg/dau (Table 1).

**TABLE 1:** Baseline characteristics of study population by quintiles of nitrate intake from the three dietary sources.

Characteristics	Categories of exposure to source‐specific nitrate intake quintile						
	Total participants	Plant‐sourced nitrate (Q1)	Plant‐sourced nitrate (Q5)	Animal‐sourced nitrate (Q1)	Animal‐sourced nitrate (Q5)	Water‐sourced nitrate (Q1)	Water‐sourced nitrate (Q5)
	(*n* = 54,804)	(*n* = 10,961)	(*n* = 10,961)	(*n* = 10,961)	(*n* = 10,961)	(*n* = 10,961)	(*n* = 10,958)
Nitrate intake from plant sources (mg/day)	44 [31, 60]	22 [18, 25]	77 [70, 88]	36 [25, 52]	53 [39, 69]	38 [27, 52]	47 [34, 64]
Nitrate intake from all animal sources (mg/day)	5.8 [4.1, 8.4]	4.6 [3.4, 6.7]	7.1 [4.7, 9.9]	3.0 [2.5, 3.4]	11.1 [9.9, 13.9]	5.6 [4.1, 8.0]	5.9 [3.9, 8.5]
Nitrate intake from drinking water (mg/day)	0.8 [0.2, 1.8]	2.9 [1.7, 4.9]	3.3 [2.0, 5.2]	3.2 [1.9, 5.2]	3.1 [1.8, 5.0]	0.0 [0.0, 0.1]	3.3 [2.6, 4.9]
**Sociodemographics**							
Age, years	56 [52, 60]	56 [53, 60]	55 [52, 60]	56 [52, 60]	56 [52, 60]	55 [52, 59]	56 [52, 60]
Sex (male)	26,062 (47.6)	5524 (50.4)	4564 (41.6)	3181 (29.0)	5836 (53.2)	7639 (69.7)	3273 (29.9)
MET score	56 [37, 85]	48 [30, 76]	64 [43, 94]	53 [34, 80]	61 [40, 91]	52 [33, 80]	61 [39, 91]
BMI (kg/m^2^)	26 [23, 28]	26 [24, 29]	25 [23, 28]	25 [23, 28]	26 [23, 28]	26 [24, 28]	26 [23, 29]
Smoking status							
Never	19,341 (35.3)	3054 (27.9)	4343 (39.6)	3925 (35.8)	4033 (36.8)	3191 (29.1)	4125 (37.6)
Former	15,676 (28.6)	2590 (23.6)	3599 (32.8)	2895 (26.4)	3272 (29.9)	3066 (28.0)	3023 (27.6)
Current	19,787 (36.1)	5317 (48.5)	3019 (27.5)	4141 (37.8)	3656 (33.4)	4704 (42.9)	3810 (34.8)
Smoking (pack‐years)	9 [0, 26]	19 [0, 32]	4 [0, 20]	8 [0, 25]	8 [0, 25]	17 [0, 33]	7 [0, 24]
*Education*							
≤7 years	10,035 (18.3)	2783 (25.4)	1444 (13.2)	2255 (20.6)	1990 (18.2)	2021 (18.4)	2346 (21.4)
8 to 11 years	29,921 (54.6)	6587 (60.1)	5141 (46.9)	6265 (57.2)	5538 (50.5)	6006 (54.8)	6192 (56.5)
≥12 years	14,848 (27.1)	1591 (14.5)	4376 (39.9)	2441 (22.3)	3433 (31.3)	2934 (26.8)	2420 (22.1)
Marital status (single)	14,184 (25.9)	3518 (32.1)	2981 (27.2)	3316 (30.3)	3074 (28.0)	2527 (23.1)	3511 (32.0)
**Comorbidities**							
CVD	2617 (4.8)	629 (5.7)	460 (4.2)	517 (4.7)	543 (5.0)	532 (4.9)	542 (4.9)
CKD	191 (0.3)	36 (0.3)	32 (0.3)	36 (0.3)	44 (0.4)	30 (0.3)	47 (0.4)
COPD	2216 (4.0)	584 (5.3)	362 (3.3)	468 (4.3)	501 (4.6)	395 (3.6)	558 (5.1)
Type 2 diabetes	550 (1.0)	91 (0.8)	144 (1.3)	121 (1.1)	90 (0.8)	85 (0.8)	151 (1.4)
Hypertension	2316 (4.2)	514 (4.7)	421 (3.8)	492 (4.5)	495 (4.5)	322 (2.9)	540 (4.9)
**Dietary intake**							
Energy	9501 [7858, 11,376]	8148 [6689, 9784]	10,768 [9090, 12,797]	7511 [6381, 8837]	11263 [9575, 13,305]	9829 [8172, 11,690]	9147 [7549, 11,003]
Vegetable (g/day)	310 [232, 403]	183 [143, 223]	475 [404, 565]	259 [187, 347]	360 [278, 459]	290 [216, 377]	324 [240, 425]
Fruit (g/day)	170 [93, 280]	87 [39, 153]	283 [177, 432]	143 [70, 249]	217 [130, 344]	132 [63, 228]	184 [105, 296]
Wholegrain (g/day)	122 [81, 169]	103 [66, 147]	153 [104, 204]	109 [68, 163]	137 [101, 188]	117 [70, 165]	125 [83, 170]
Red meat (g/day)	81 [59, 111]	75 [55, 101]	82 [56, 116]	56 [42, 70]	104 [75, 141]	93 [68, 123]	73 [52, 99]
Poultry (g/day)	18 [10, 28]	13 [7, 20]	23 [13, 35]	13 [7, 21]	21 [13, 33]	17 [9, 26]	18 [10, 29]
Processed meat (g/day)	22 [12, 36]	22 [13, 37]	19 [10, 33]	14 [8, 23]	26 [15, 43]	26 [15, 41]	19 [10, 31]
Fish (g/day)	38 [25, 55]	29 [19, 42]	48 [32, 68]	29 [19, 41]	48 [32, 68]	36 [23, 52]	39 [26, 58]
Dairy (g/day)	306 [166, 571]	263 [120, 547]	354 [215, 616]	139 [76, 287]	541 [382, 797]	289 [145, 564]	303 [164, 560]
Sugar and confectionery (g/day)	49 [28, 81]	42 [23, 73]	53 [32, 88]	41 [22, 70]	57 [34, 93]	49 [28, 85]	46 [26, 76]
Tea (g/day)	86 [3, 500]	16 [0, 200]	200 [16, 500]	86 [3, 500]	157 [16, 500]	16 [3, 500]	86 [7, 500]
Coffee (g/day)	900 [500, 1300]	900 [500, 1300]	500 [500, 900]	900 [500, 1300]	900 [500, 1300]	900 [500, 1300]	900 [500, 900]
Soft drink (g/day)	16 [3, 45]	16 [3, 86]	7 [0, 29]	7 [0, 29]	16 [3, 57]	16 [3,86]	10 [0, 29]
Vegetable oils (g/day)	5 [1, 9]	1 [1, 3]	9 [5, 13]	2 [1, 6]	5 [2, 11]	3 [1, 8]	5 [1, 9]
Alcohol (g/day)	13 [6, 31]	12 [4, 32]	13 [6, 29]	11 [3, 25]	13 [6, 31]	16 [7, 37]	11 [4, 23]
Polyphenols (mg/day)	1606 [1265, 1948]	1425 [1089, 1764]	1768 [1431, 2154]	1516 [1158, 1859]	1683 [1348, 2056]	1631 [1271, 1952]	1590 [1233, 1963]
Vitamin C (mg/day)	125 [86, 184]	74 [52, 116]	185 [141, 260]	110 [71, 168]	150 [108, 214]	105 [73, 153]	140 [96, 215]
Vitamin E (mg α‐TE/day)	12 [8, 18]	9 [6, 14]	15 [11, 22]	10 [6, 16]	14 [10, 20]	11 [8, 16]	13 [9, 19]
Folic acid (µg/day)	367 [289, 462]	273 [218, 349]	473 [398, 567]	299 [234, 387]	446 [364, 543]	343 [270, 432]	382 [298, 485]

*Note*: Data expressed as median [IQR] or *n* (%), unless otherwise stated.

Abbreviations: BMI, body mass index; CVD, cardiovascular disease, CKD, chronic kidney disease; COPD, common obstructive pulmonary disease; MET, metabolic equivalent of task (determined from physical activity questionnaire).

### 3.1 Baseline characteristics

Participants in the highest quintile of plant‐sourced nitrate intake, compared to participants in the lowest quintile of plant‐sourced nitrate intake, were more likely to be female, have a higher level of physical activity, never have smoked, have more years of education, be living with a partner, and have a higher total energy intake (Table 1). They were also less likely to have CVD and COPD but more likely to have diabetes. For total animal‐sourced nitrate intake, those with higher intakes compared to those with lowest intakes were more likely to be male and have a higher total energy intake. For water‐sourced nitrate, participants in the highest quintile were more likely to be female, have a higher level of physical activity, be smokers, have a lower number of years of education, and be single.

### 3.2 Associations between source‐dependent nitrate and nitrite intakes and incident dementia

**FIGURE 1:**
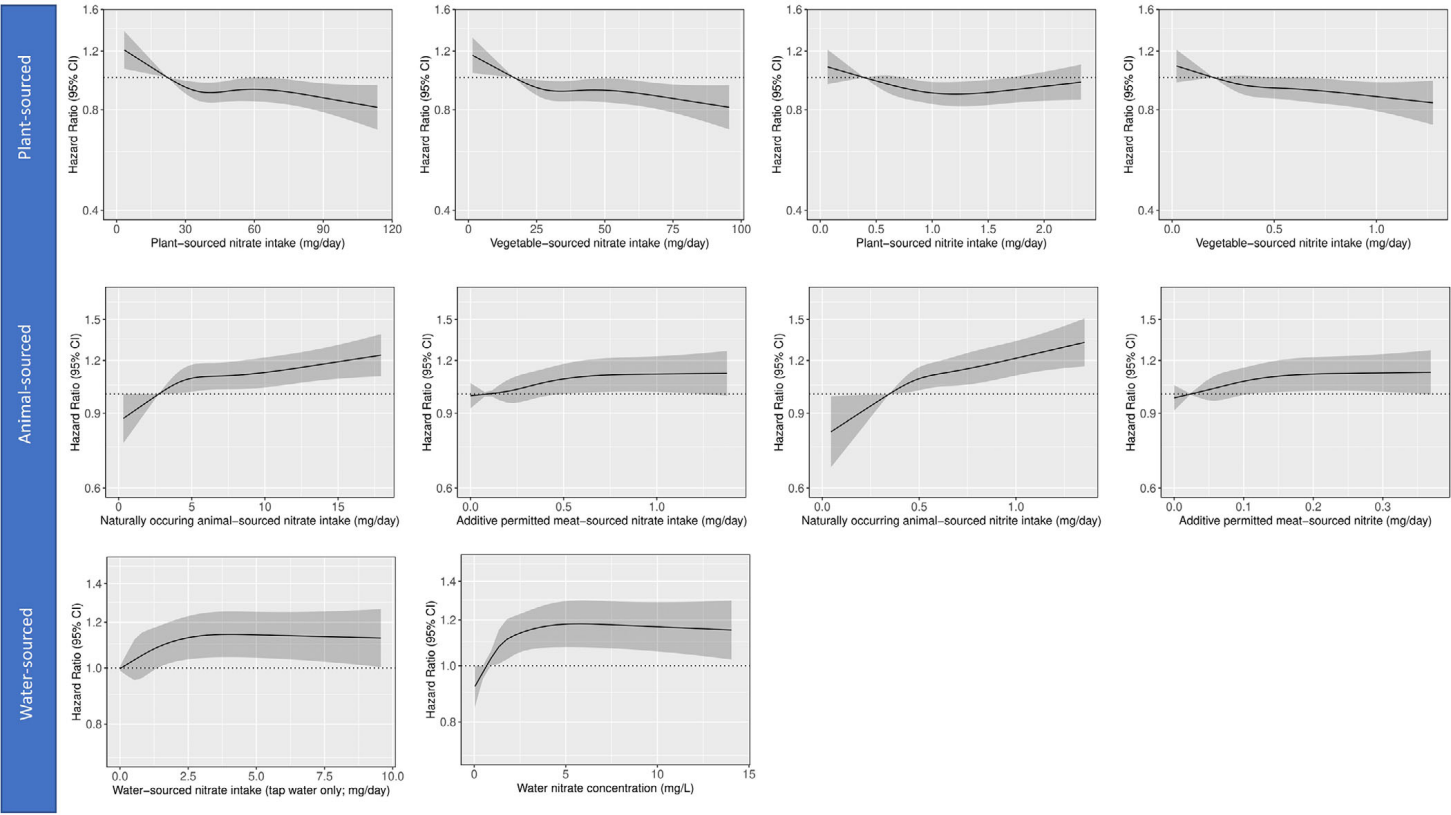
Cubic spline curves depicting association between source‐dependent nitrate and nitrite intakes and incident dementia in participants of Danish Diet, Cancer and Health Cohort Study (*n* = 54,804). Hazard ratios and 95% confidence intervals (CIs) are based on Cox proportional hazards models and compare the specific level of the exposure (horizontal axis) to the median intake for participants in the lowest intake quintile. Associations are adjusted for age, sex, body mass index, smoking status, smoking pack‐years, alcohol consumption, education level, physical activity level, living situation, intakes of (1) red meat, processed meat, poultry, dairy, fish, sugar and confections, soft drinks, refined grains, coffee, and tea when plant‐sourced nitrate or nitrite was the exposure of interest, (2) whole grains, refined grains, vegetables, fruits, vegetable oils, sugar and confections, soft drinks, refined grains, coffee, and tea when animal‐sourced nitrate or nitrite was the exposure of interest, and (3) whole grains, refined grains, red meat, processed meat, poultry, dairy, fish, vegetables, fruits, vegetable oils, sugar and confections, soft drinks, coffee, and tea when water‐sourced nitrate was the exposure of interest. Additionally, tap water intake was included as a covariate when water nitrate concentration was the exposure of interest.

Restricted cubic splines show a non‐linear inverse association between intakes of both plant‐sourced and vegetable‐sourced nitrate and incident dementia (Figure 1). Compared to those in the lowest intake quintile, participants in the highest intake quintiles of plant‐sourced and vegetable‐sourced nitrate had a 10% (fifth quintile vs first quintile hazard ratio [HR_Q5vsQ1_]: 0.90 [0.83, 0.98]) and 11% (HR_Q5vsQ1_: 0.89 [0.82, 0.97]) lower rate of dementia, respectively, while a doubling in intake of plant‐ and vegetable‐sourced nitrate was each associated with an 8% lower rate of dementia, after adjusting for demographic, lifestyle, and dietary confounders (Model 3; Table 2). Very similar associations were seen for intakes of plant‐sourced nitrite (HR_Q5vsQ1_: 0.90 [0.82, 0.98]) and vegetable‐sourced nitrite (HR_Q5vsQ1_: 0.90 [0.82, 0.98]).

**TABLE 2:** Hazard ratio of dementia by quintiles of source‐dependent nitrate and nitrite intakes.

	Q1	Q2	Q3	Q4	Q5	
	*n* = 10,961	*n* = 10,961	*n* = 10,960	*n* = 10,961	*n* = 10,961	Log_2_
**Plant‐sourced nitrate**						
Intake (mg/day)	22 [18, 25]	34 [31, 36]	44 [41, 47]	56 [53, 60]	77 [70, 88]	
No. events	984	931	977	925	933	
Model 1	Ref.	0.89 (0.84, 0.94)	0.87 (0.82, 0.93)	0.88 (0.82, 0.95)	0.88 (0.82, 0.95)	0.92 (0.88, 0.96)
Model 2	Ref.	0.92 (0.87, 0.98)	0.92 (0.86, 0.98)	0.95 (0.88, 1.02)	0.94 (0.87, 1.02)	0.95 (0.91, 0.99)
Model 3	Ref.	0.91 (0.86, 0.97)	0.90 (0.84, 0.97)	0.92 (0.85, 1.00)	0.90 (0.83, 0.98)	0.92 (0.88, 0.96)
**Vegetable‐sourced nitrate**						
Intake (mg/day)	17 [13, 19]	26 [24, 29]	35 [33, 38]	46 [43, 49]	64 [58, 74]	
No. events	999	958	931	973	889	
Model 1	Ref.	0.91 (0.86, 0.96)	0.88 (0.82, 0.94)	0.88 (0.82, 0.95)	0.87 (0.81, 0.94)	0.92 (0.89, 0.96)
Model 2	Ref.	0.93 (0.88, 0.99)	0.92 (0.86, 0.99)	0.94 (0.87, 1.01)	0.93 (0.86, 1.00)	0.94 (0.91, 0.98)
Model 3	Ref.	0.93 (0.87, 0.98)	0.91 (0.85, 0.97)	0.92 (0.85, 0.99)	0.89 (0.82, 0.97)	0.92 (0.89, 0.96)
**Plant‐sourced nitrite**						
Intake (mg/day)	0.4 [0.3, 0.5]	0.6 [0.6, 0.7]	0.8 [0.7, 0.9]	1.0 [1.0, 1.1]	1.5 [1.3, 1.7]	
No. events	997	965	913	922	953	
Model 1	Ref.	0.92 (0.87, 0.98)	0.88 (0.82, 0.94)	0.86 (0.80, 0.92)	0.87 (0.81, 0.94)	0.94 (0.90, 0.98)
Model 2	Ref.	0.96 (0.90, 1.01)	0.93 (0.87, 1.00)	0.92 (0.85, 0.98)	0.93 (0.86, 1.01)	0.97 (0.92, 1.01)
Model 3	Ref.	0.95 (0.90, 1.01)	0.92 (0.86, 0.99)	0.90 (0.83, 0.97)	0.90 (0.82, 0.98)	0.94 (0.90, 0.99)
**Vegetable‐sourced nitrite**						
Intake (mg/day)	0.2 [0.2, 0.2]	0.3 [0.3, 0.4]	0.5 [0.4, 0.5]	0.6 [0.6, 0.6]	0.8 [0.8, 1.0]	
No. events	1056	971	911	955	857	
Model 1	Ref.	0.93 (0.88, 0.98)	0.90 (0.84, 0.96)	0.89 (0.83, 0.96)	0.88 (0.82, 0.95)	0.92 (0.88, 0.97)
Model 2	Ref.	0.96 (0.91, 1.02)	0.95 (0.88, 1.01)	0.95 (0.88, 1.02)	0.93 (0.86, 1.01)	0.95 (0.91, 0.99)
Model 3	Ref.	0.95 (0.90, 1.01)	0.93 (0.87, 1.00)	0.92 (0.85, 1.00)	0.90 (0.82, 0.98)	0.92 (0.88, 0.97)
**Naturally occurring animal‐sourced nitrate**						
Intake (mg/day)	2.7 [2.3, 3.1]	4.0 [3.7, 4.3]	5.4 [5.0, 5.9]	7.5 [6.9, 8.1]	10.7 [9.5, 13.5]	
No. events	889	950	910	970	1031	
Model 1	Ref.	1.02 (0.97, 1.08)	1.03 (0.96, 1.10)	1.02 (0.95, 1.10)	1.05 (0.97, 1.13)	1.04 (1.00, 1.08)
Model 2	Ref.	1.03 (0.98, 1.09)	1.05 (0.97, 1.12)	1.05 (0.98, 1.13)	1.08 (1.00, 1.17)	1.05 (1.01, 1.08)
Model 3	Ref.	1.06 (1.00, 1.12)	1.10 (1.02, 1.18)	1.10 (1.03, 1.19)	1.13 (1.04, 1.23)	1.05 (1.01, 1.09)
**Additive permitted meat‐sourced nitrate**						
Intake (mg/day)	0.1 [0.1, 0.1]	0.2 [0.2, 0.2]	0.3 [0.3, 0.3]	0.4 [0.4, 0.5]	0.7 [0.6, 0.9]	
No. events	996	958	911	939	946	
Model 1	Ref.	0.99 (0.94, 1.04)	1.01 (0.94, 1.08)	1.06 (0.99, 1.14)	1.13 (1.04, 1.23)	1.06 (1.02, 1.10)
Model 2	Ref.	0.99 (0.94, 1.05)	1.01 (0.94, 1.08)	1.05 (0.97, 1.13)	1.09 (1.00, 1.19)	1.04 (1.00, 1.08)
Model 3	Ref.	1.01 (0.96, 1.07)	1.03 (0.96, 1.12)	1.07 (0.99, 1.16)	1.11 (1.01, 1.22)	1.03 (0.99, 1.07)
**Naturally occurring animal‐sourced nitrite**						
Intake (mg/day)	0.3 [0.3, 0.4]	0.5 [0.4, 0.5]	0.6 [0.5, 0.6]	0.7 [0.6,0.7]	0.9 [0.8, 1.1]	
No. events	917	914	957	959	1003	
Model 1	Ref.	1.03 (0.97, 1.08)	1.04 (0.98, 1.11)	1.07 (0.99, 1.15)	1.13 (1.04, 1.23)	1.13 (1.07, 1.21)
Model 2	Ref.	1.04 (0.98, 1.09)	1.05 (0.99, 1.12)	1.06 (0.99, 1.14)	1.11 (1.02, 1.21)	1.11 (1.04, 1.18)
Model 3	Ref.	1.07 (1.01, 1.13)	1.11 (1.03, 1.18)	1.13 (1.05, 1.22)	1.19 (1.08, 1.30)	1.12 (1.05, 1.20)
**Additive permitted meat‐sourced nitrite**						
Intake (mg/day)	0.0 [0.0, 0.0]	0.0 [0.0, 0.1]	0.1 [0.1, 0.1]	0.1 [0.1, 0.1]	0.2 [0.2, 0.2]	
No. events	981	951	958	945	915	
Model 1	Ref.	1.00 (0.95, 1.06)	1.03 (0.96, 1.10)	1.08 (1.01, 1.16)	1.15 (1.05, 1.25)	1.12 (1.05, 1.19)
Model 2	Ref.	1.00 (0.95, 1.06)	1.02 (0.95, 1.10)	1.06 (0.98, 1.14)	1.10 (1.01, 1.20)	1.08 (1.01, 1.15)
Model 3	Ref.	1.02 (0.97, 1.08)	1.05 (0.97, 1.13)	1.08 (1.00, 1.17)	1.11 (1.02, 1.22)	1.06 (1.00, 1.14)
**Tap water‐sourced nitrate**						
Intake (mg/day)	0 [0, 0.1]	0.3 [0.2, 0.4]	0.8 [0.6, 0.9]	1.5 [1.1, 1.8]	3.3 [2.6, 4.9]	
No. events	880	862	983	1014	1011	
Model 1	Ref.	0.98 (0.92, 1.04)	0.98 (0.90, 1.08)	1.03 (0.95, 1.12)	1.09 (1.00, 1.19)	1.01 (1.00, 1.03)
Model 2	Ref.	1.00 (0.95, 1.06)	1.02 (0.93, 1.11)	1.05 (0.97, 1.14)	1.09 (1.00, 1.19)	1.01 (1.00, 1.03)
Model 3	Ref.	1.02 (0.96, 1.09)	1.06 (0.96, 1.16)	1.10 (1.01, 1.19)	1.14 (1.05, 1.25)	1.02 (1.00, 1.04)

*Note*: Hazard ratios (HRs) and 95% confidence intervals (CIs) for total dementia during up to 27 years of follow‐up, obtained from Cox proportional hazards models. HRs and 95% CIs for each quintile (30th, 50th, 70th, and 90th percentiles) were derived from restricted cubic spline models, with the 10th percentile as the reference. Log_2_‐transformed HRs represent per doubling of the exposure. Model 1 included age and sex; Model 2 included age, sex, body mass index, smoking status, smoking packyears, alcohol consumption, education level, physical activity level, and living situation; Model 3 adjusted for the covariates in Model 2 plus intakes of (1) red meat, processed meat, poultry, dairy, fish, sugar and confections, soft drinks, refined grains, coffee, and tea when plant‐sourced nitrate or nitrite was the exposures of interest, (2) whole grains, refined grains, vegetables, fruits, vegetable oils, sugar and confections, soft drinks, refined grains, coffee, and tea when animal‐sourced nitrate or nitrite were the exposures of interest, and (3) whole grains, refined grains, red meat, processed meat, poultry, dairy, fish, vegetables, fruits, vegetable oils, sugar and confections, and soft drinks when water‐sourced nitrate was the exposure of interest.

Exposure intakes are presented as median (interquartile range [IQR]).

Both naturally occurring animal‐sourced and additive‐permitted meat‐sourced nitrate intakes were associated with incident dementia (Figure 1). Participants in the highest naturally occurring animal‐sourced and additive‐permitted meat‐sourced nitrate intake quintiles had a 13% (HR_Q5vsQ1_: 1.13 [1.04, 1.23]) and 11% (HR_Q5vsQ1_: 1.11 [1.01, 1.22]) higher rate of dementia, respectively, compared to those in the lowest intake quintiles (Model 3; Table 2). Comparable associations were seen for intakes of naturally occurring animal‐sourced nitrite (HR_Q5vsQ1_: 1.19 [1.08, 1.30]) and additive‐permitted meat‐sourced nitrite (HR_Q5vsQ1_: 1.11 [1.02, 1.22]).

A higher consumption of nitrate from tap water at baseline was non‐linearly associated with a higher rate of dementia (Figure 1), whereby participants in the highest intake quintile had a 14% higher rate of dementia (HR_Q5vsQ1_: 1.14 [1.05, 1.25]; Table 2) compared to those in the lowest intake quintile. Comparable associations were observed when modeling baseline water nitrate concentration adjusted for baseline tap water intake (HR_Q5vsQ1_: 1.18 [1.08, 1.29]; Figure S2). When modeling the water nitrate exposure as a time‐varying covariate, averaged over a 15‐year window, drinking water nitrate concentration was associated with all incident dementia (Table S2). Compared to participants consuming drinking water with the lowest nitrate concentration (Q1, mean: 0.8 mg/L), those consuming water with higher nitrate concentrations (Q3–Q5, mean: 1.8–5.1 mg/L) had a 12% to 16% higher rate of dementia, after adjusting for demographic, lifestyle, and dietary confounders.

Comparable trends were seen when the cumulative incidence of dementia by quintiles of nitrate exposures was plotted using the Aalen–Johansen estimator, accounting for death as a competing risk (Figure S3).

### 3.3 Associations between source‐dependent nitrate and nitrite intakes and early‐onset dementia

For all exposures, associations with early‐onset dementia were more pronounced (Table 3). The highest intakes of plant‐sourced and vegetable‐sourced nitrate were associated with a 31% (HR_Q5vsQ1_: 0.69 [0.44, 1.08]) and 39% (HR_Q5vsQ1_: 0.61 [0.39, 0.95]) lower rate of early‐onset dementia, while the highest intakes of naturally occurring animal‐sourced, additive‐permitted meat‐sourced, and tap water‐sourced nitrate were associated with a 73% (HR_Q5vsQ1_: 1.73 [1.12, 2.66]), 40% (HR_Q5vsQ1_: 1.40 [0.87, 2.25]), and 53% (HR_Q5vsQ1_: 1.53 [0.99, 2.37]) higher rate of early‐onset dementia, respectively. In general, associations between each of the exposures and incident early‐onset dementia appeared to weaken (HRs became closer to 1) as the age of dementia diagnosis increased (Table S3).

**TABLE 3:** Hazard ratio of early‐onset dementia by quintiles of source‐dependent nitrate and nitrite intakes.

	Q1	Q2	Q3	Q4	Q5	
	*n* = 10,961	*n* = 10,961	*n* = 10,960	*n* = 10,961	*n* = 10,961	Log_2_
**Plant‐sourced nitrate**						
Intake (mg/day)	22 [18, 25]	34 [31, 36]	44 [41, 47]	56 [53, 60]	77 [70, 88]	
No. events	62	48	37	39	33	
Model 1	Ref.	0.72 (0.57, 0.91)	0.64 (0.49, 0.83)	0.62 (0.44, 0.87)	0.55 (0.38, 0.81)	0.69 (0.57, 0.83)
Model 2	Ref.	0.80 (0.63, 1.02)	0.75 (0.57, 0.99)	0.74 (0.52, 1.06)	0.67 (0.44, 1.00)	0.75 (0.62, 0.92)
Model 3	Ref.	0.87 (0.68, 1.12)	0.83 (0.62, 1.13)	0.81 (0.55, 1.20)	0.69 (0.44, 1.08)	0.78 (0.63, 0.95)
**Vegetable‐sourced nitrate**						
Intake (mg/day)	17 [13, 19]	26 [24, 29]	35 [33, 38]	46 [43, 49]	64 [58, 74]	
No. events	62	45	43	38	31	
Model 1	Ref.	0.64 (0.51, 0.81)	0.57 (0.44, 0.74)	0.59 (0.42, 0.82)	0.50 (0.34, 0.73)	0.64 (0.55, 0.74)
Model 2	Ref.	0.71 (0.56, 0.89)	0.66 (0.50, 0.86)	0.68 (0.47, 0.97)	0.58 (0.38, 0.87)	0.68 (0.58, 0.79)
Model 3	Ref.	0.77 (0.60, 0.98)	0.73 (0.54, 0.98)	0.74 (0.51, 1.10)	0.61 (0.39, 0.95)	0.69 (0.58, 0.82)
**Plant‐sourced nitrite**						
Intake (mg/day)	0.4 [0.3, 0.5]	0.6 [0.6, 0.7]	0.8 [0.7, 0.9]	1.0 [1.0, 1.1]	1.5 [1.3, 1.7]	
No. events	57	55	29	29	49	
Model 1	Ref.	0.74 (0.58, 0.96)	0.62 (0.46, 0.82)	0.55 (0.40, 0.76)	0.58 (0.40, 0.85)	0.78 (0.63, 0.97)
Model 2	Ref.	0.84 (0.65, 1.09)	0.74 (0.55, 1.00)	0.67 (0.48, 0.95)	0.71 (0.47, 1.06)	0.86 (0.69, 1.08)
Model 3	Ref.	0.94 (0.72, 1.23)	0.85 (0.61, 1.18)	0.75 (0.52, 1.10)	0.76 (0.49, 1.18)	0.91 (0.72, 1.15)
**Vegetable‐sourced nitrite**						
Intake (mg/day)	0.2 [0.2, 0.2]	0.3 [0.3, 0.4]	0.5 [0.4, 0.5]	0.6 [0.6, 0.6]	0.8 [0.8, 1.0]	
No. events	61	41	47	36	34	
Model 1	Ref.	0.77 (0.59, 0.99)	0.66 (0.49, 0.88)	0.60 (0.43, 0.86)	0.50 (0.34, 0.74)	0.64 (0.51, 0.80)
Model 2	Ref.	0.86 (0.66, 1.11)	0.78 (0.57, 1.06)	0.72 (0.50, 1.04)	0.59 (0.39, 0.89)	0.69 (0.55, 0.87)
Model 3	Ref.	0.96 (0.73, 1.26)	0.90 (0.65, 1.27)	0.82 (0.55, 1.23)	0.66 (0.42, 1.04)	0.73 (0.57, 0.95)
**Naturally occurring animal‐sourced nitrate**						
Intake (mg/day)	2.7 [2.3, 3.1]	4.0 [3.7, 4.3]	5.4 [5.0, 5.9]	7.5 [6.9, 8.1]	10.7 [9.5, 13.5]	
No. events	35	43	45	40	56	
Model 1	Ref.	1.07 (0.80, 1.44)	1.15 (0.77, 1.70)	1.23 (0.84, 1.80)	1.30 (0.87, 1.94)	1.15 (0.95, 1.38)
Model 2	Ref.	1.15 (0.85, 1.54)	1.28 (0.87, 1.90)	1.41 (0.96, 2.06)	1.49 (0.99, 2.24)	1.19 (0.99, 1.44)
Model 3	Ref.	1.28 (0.95, 1.73)	1.52 (1.01, 2.27)	1.66 (1.11, 2.49)	1.73 (1.12, 2.66)	1.22 (1.00, 1.48)
**Additive permitted meat‐sourced nitrate**						
Intake (mg/day)	0.1 [0.1, 0.1]	0.2 [0.2, 0.2]	0.3 [0.3, 0.3]	0.4 [0.4, 0.5]	0.7 [0.6, 0.9]	
No. events	35	37	43	45	59	
Model 1	Ref.	1.04 (0.77, 1.41)	1.11 (0.73, 1.68)	1.21 (0.81, 1.80)	1.39 (0.89, 2.16)	1.25 (1.04, 1.50)
Model 2	Ref.	1.09 (0.81, 1.47)	1.16 (0.77, 1.76)	1.22 (0.82, 1.82)	1.33 (0.85, 2.09)	1.21 (1.01, 1.45)
Model 3	Ref.	1.14 (0.84, 1.55)	1.25 (0.82, 1.90)	1.30 (0.86, 1.97)	1.40 (0.87, 2.25)	1.22 (1.01, 1.47)
**Naturally occurring animal‐sourced nitrite**						
Intake (mg/day)	0.3 [0.3, 0.4]	0.5 [0.4, 0.5]	0.6 [0.5, 0.6]	0.7 [0.6,0.7]	0.9 [0.8, 1.1]	
No. events	39	41	41	44	54	
Model 1	Ref.	1.08 (0.81, 1.44)	1.08 (0.77, 1.52)	1.03 (0.71, 1.50)	1.02 (0.66, 1.56)	1.04 (0.76, 1.43)
Model 2	Ref.	1.15 (0.87, 1.54)	1.17 (0.83, 1.65)	1.11 (0.76, 1.62)	1.06 (0.69, 1.64)	1.05 (0.76, 1.44)
Model 3	Ref.	1.24 (0.91, 1.69)	1.39 (0.90, 2.14)	1.43 (0.93, 2.18)	1.50 (0.93, 2.42)	1.08 (0.77, 1.52)
**Additive permitted meat‐sourced nitrite**						
Intake (mg/day)	0.0 [0.0, 0.0]	0.0 [0.0, 0.1]	0.1 [0.1, 0.1]	0.1 [0.1, 0.1]	0.2 [0.2, 0.2]	
No. events	33	32	48	47	59	
Model 1	Ref.	1.12 (0.82, 1.53)	1.23 (0.80, 1.88)	1.33 (0.89, 1.99)	1.49 (0.96, 2.32)	1.51 (1.16, 1.97)
Model 2	Ref.	1.17 (0.86, 1.60)	1.29 (0.84, 1.98)	1.35 (0.90, 2.03)	1.45 (0.92, 2.29)	1.45 (1.11, 1.90)
Model 3	Ref.	1.01 (0.48, 2.12)	1.07 (0.36, 3.21)	1.21 (0.38, 3.85)	1.37 (0.38, 4.96)	1.46 (1.11, 1.93)
**Tap water‐sourced nitrate intake**						
Intake (mg/day)	0 [0, 0.1]	0.3 [0.2, 0.4]	0.8 [0.6, 0.9]	1.5 [1.1, 1.8]	3.3 [2.6, 4.9]	
No. events	43	45	44	35	52	
Model 1	Ref.	1.03 (0.78, 1.36)	1.08 (0.70, 1.65)	1.13 (0.77, 1.66)	1.23 (0.80, 1.88)	1.06 (0.97, 1.16)
Model 2	Ref.	1.12 (0.85, 1.48)	1.23 (0.80, 1.89)	1.27 (0.87, 1.86)	1.33 (0.87, 2.05)	1.08 (0.99, 1.17)
Model 3	Ref.	1.20 (0.91, 1.59)	1.39 (0.90, 2.14)	1.45 (0.98, 2.13)	1.53 (0.99, 2.37)	1.09 (1.00, 1.19)

*Note*: Hazard ratios (HRs) and 95% confidence intervals (CIs) for early‐onset dementia during up to 27 years of follow‐up, obtained from Cox proportional hazards models. HRs for each quintile (30th, 50th, 70th, and 90th percentiles) were derived from restricted cubic spline models, with the 10th percentile as the reference. Log_2_‐transformed HRs represent per doubling of the exposure Model 1 included age and sex; Model 2 included age, sex, body mass index, smoking status, smoking pack‐years, alcohol consumption, education level, physical activity level and living situation; Model 3 adjusted for the covariates in Model 2 plus intakes of (1) red meat, processed meat, poultry, dairy, fish, sugar and confections, soft drinks, refined grains, coffee, and tea when plant‐sourced nitrate or nitrite was the exposure of interest, (2) whole grains, refined grains, vegetables, fruits, vegetable oils, sugar and confections, soft drinks, refined grains, coffee, and tea when animal‐sourced nitrate or nitrite was the exposure of interest, and (3) whole grains, refined grains, red meat, processed meat, poultry, dairy, fish, vegetables, fruits, vegetable oils, sugar and confections, and soft drinks when water‐sourced nitrate was the exposure of interest.

Exposure intakes are presented as median (interquartile range [IQR]).

### 3.4 Stratified analyses

**FIGURE 2:**
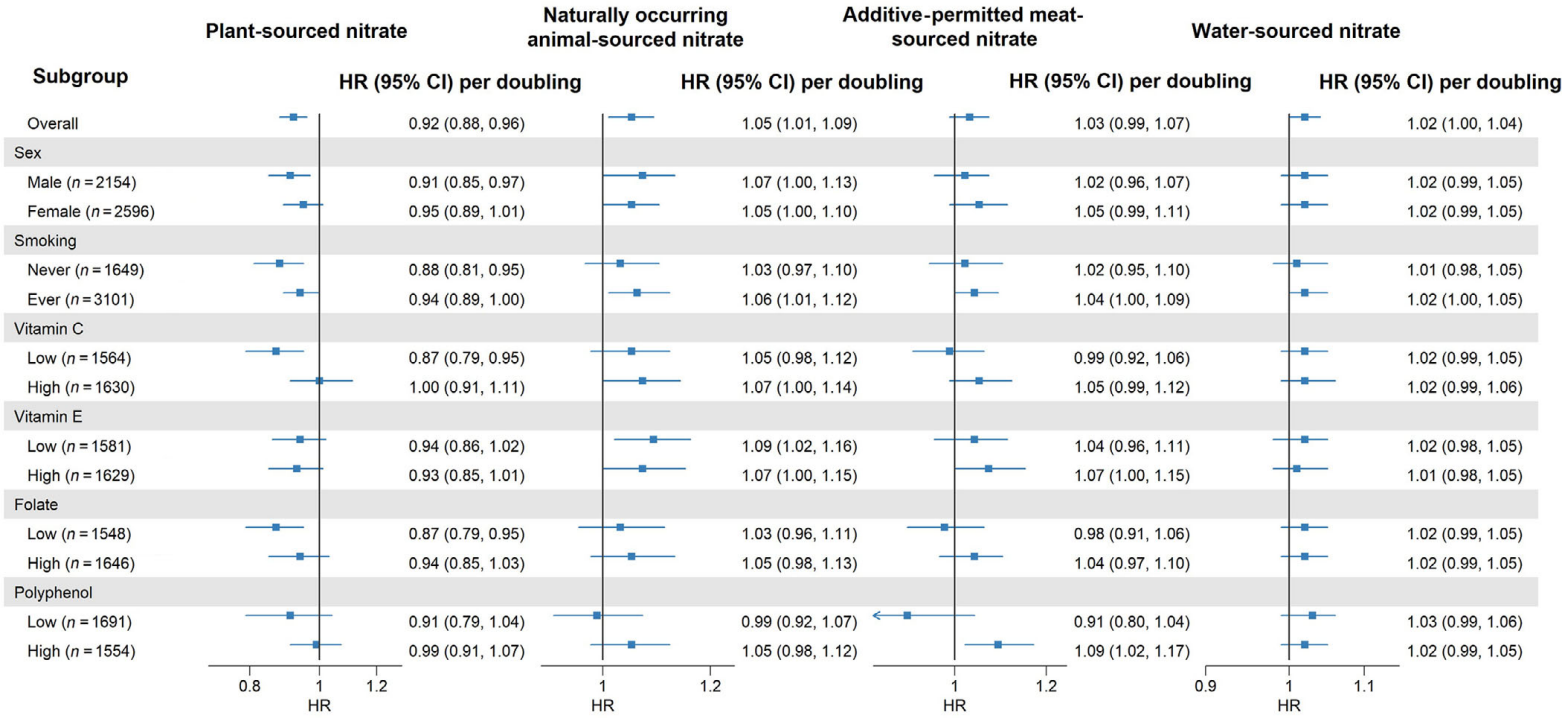
Forest plots depicting associations between plant‐sourced, natural occurring animal‐sourced, additive‐permitted meat‐sourced and tap water only‐sourced nitrate intake and incident dementia, stratified by sex, smoking status, and intakes of vitamin C, vitamin E, folate, and polyphenols. Hazard ratios and 95% confidence intervals (CIs), per doubling of the exposure (i.e., log_2_‐trasnformed), are derived from Cox proportional hazards models. All analyses were adjusted for age, sex, body mass index, smoking status, smoking pack‐years, alcohol consumption, education level, physical activity level, living situation, and (1) intakes of red meat, processed meat, poultry, dairy, fish, sugar and confections, soft drinks, refined grains, coffee, and tea when the exposure was plant‐sourced nitrate, (2) intakes of whole grains, refined grains, vegetables, fruits, vegetable oils, sugar and confections, soft drinks, refined grains, coffee, and tea when the exposure was animal‐sourced nitrate, and (3) intakes of whole grains, refined grains, red meat, processed meat, poultry, dairy, fish, vegetables, fruits, vegetable oils, sugar and confections, soft drinks, coffee, and tea when the exposure was water‐sourced nitrate (Model 3).

The lower rate of total dementia observed for high, compared to low, intakes of plant‐sourced nitrate in the whole cohort was not found in participants with a high intake of vitamin C or polyphenols (Figure 2). Evidence of a higher rate of dementia for higher intakes of naturally occurring animal‐sourced nitrate was seen across all subgroups, except low polyphenol consumers. For additive‐permitted meat‐sourced nitrate, a higher rate of dementia was only seen in high polyphenol consumers. Associations between water‐sourced nitrate and dementia were fairly constant across all subgroups.

### 3.5 Sensitivity analyses

When restricting the follow‐up period to 10 years, associations between all exposures and incident dementia were strengthened (e.g., HR_Q5vsQ1_: plant‐sourced nitrate 0.65 [0.49, 0.87], vegetable‐sourced nitrate: 0.57 [0.43, 0.76], naturally occurring‐animal sourced‐nitrate: 1.37 [1.04, 1.80], water‐sourced nitrate: 1.31 [0.98, 1.75]), except additive‐permitted meat‐sourced nitrate, and nitrite for which associations became null (Table S4).

## 4 DISCUSSION

In this prospective cohort of 54,804 dementia‐free Danish adults followed for 27 years, higher intakes of plant‐ and vegetable‐sourced nitrate and nitrite were associated with a 10% to 11% lower total and 24% to 34% lower early‐onset dementia rates. Conversely, higher intakes of animal‐sourced nitrate and nitrite were associated with 11% to 19% higher total and 73% higher early‐onset dementia rates. Additive‐permitted meat‐sourced nitrate and nitrite were associated with a 11% higher total dementia rate and tap water‐sourced nitrate with a 14% higher rate. No consistent patterns were observed when associations were stratified by factors hypothesized to influence *N*‐nitrosamine formation.

Consistent with the hypothesis that nitrate's benefits depend on co‐ingested polyphenols and antioxidant vitamins, higher plant‐sourced nitrate, and nitrite was associated with lower total and early‐onset dementia rates. Lower rates were observed at moderate to high intake levels (median 34 to 77 mg/day nitrate, 0.6 to 1.5 mg/day nitrite), which was derived mainly from vegetables, particularly lettuce and potatoes,^36^ and equates to approximately 0.5 to 1.5 cups of raw green leafy vegetables daily. Potatoes, though low in nitrate, contributed significantly due to high consumption. Although this is the first study to examine associations of plant‐, animal‐, and drinking water‐sourced nitrite with dementia, our findings for vegetable‐sourced nitrate align with two recent studies. In 9543 Rotterdam Study participants, each 50‐mg/day increment in vegetable nitrate was associated with a 14% lower dementia risk.^44^ Similarly, among 9149 Australian Diabetes, Obesity, and Lifestyle (AusDiab) study participants, plant‐ (median intake 98 mg/day) and vegetable‐sourced nitrate (median intake 72 mg/day) had a 57% and 66% lower risk of dementia‐related mortality, respectively.^20^ In the present study, participants with the highest vegetable‐sourced nitrate intake had an 11% lower dementia rate, strengthening to 43% when follow‐up was restricted to 10 years, indicating possible attenuation from exposure misclassification over longer follow‐up. Although lifestyle factors such as smoking, hypertension, and education remain dominant dementia risk determinants, nitrate source, particularly from vegetables, appears a meaningful, modifiable contributor within the broader context of diet quality and vascular health.

The association between plant‐sourced nitrate and dementia is biologically plausible through the entero‐salivary nitrate–nitrite–NO pathway, whereby nitrate serves as a precursor for NO,^3^ a key regulator of vascular and neuronal function.^10^, ^11^ Clinical trials show nitrate intake enhances NO‐mediated vascular outcomes,^45^ effects that diminish when this pathway is disrupted.^46^, ^47^ While other bioactives such as flavonoids are also linked to lower dementia risk^48^ with potential overlapping or synergistic effects, these findings highlight plant nitrate as a potential contributor to neuroprotection. Whether neuroprotection arises via vascular or dementia‐specific mechanisms remains to be determined. Given diet's complexity, isolating nitrate's influence from co‐consumed nutrients is challenging. Long‐term clinical trials integrating cognitive assessments and dementia biomarkers are needed to confirm causality and define nitrate's mechanistic role within broader dietary patterns.

This study supports the hypothesis that nitrate's adverse effects are source dependent, with higher total and early‐onset dementia rates observed for animal‐sourced nitrate and nitrite. Elevated risk was seen in the top intake quintiles (∼4 to 11 mg/day nitrate; 0.5 to 0.9 mg/day nitrite), equivalent to ∼130 to 350 g/day beef or 160 to 440 g/day yoghurt,^36^ though *N*‐nitrosamine formation likely differs due to yoghurt's low amine content and lack of heme. Intakes from naturally occurring animal sources were ∼10‐fold higher than from meat containing permitted additives. Fresh meats, particularly red meats, and dairy are significant, though under‐recognized, nitrate and nitrite sources.^37^ Prior studies on meat intake and dementia show inconsistent results –lower,^49^, ^50^ higher,^51^ or no associations.^52^, ^53^ Despite low nitrite additive intakes (median 0.2 mg/day nitrite, ∼30 g bacon), associations with higher dementia rates were observed. Prior studies are also inconsistent with higher^49^, ^51^ or no associations^54^ observed. A possible mechanism involves *N*‐nitrosamines formation, given their neurotoxic potential.^15^, ^55^ Contributing factors to *N*‐nitrosamine formation include the presence of amines, high‐heat cooking methods, smoking processes, and lack of nitrosation inhibitors.^37^ Other compounds in meat such as polycyclic and heterocyclic aromatic amines may also contribute,^56^ although their effects are difficult to distinguish due to correlations with nitrate/nitrite. These findings support recommendations for a more plant‐based diet to promote brain health.

This study further supports the hypothesis that nitrate's health effects vary by source, with higher dementia rates observed for the highest quintile of tap water‐sourced nitrate intake compared to lower intakes. This association persisted when modeling both baseline and time‐updated tap water nitrate concentrations. Increased agricultural activities, use of ammonia‐rich fertilizers, nitrogen‐fixing crops, and fossil fuel combustion have doubled nitrate deposition, raising water concentrations and prompting global regulatory limits of 44 to 50 mg/L.^16^ Notably, higher dementia rates were observed for median 15‐year average drinking water nitrate levels of 1.8 to 5.1 mg/L – levels well below these regulatory thresholds. Although drinking water nitrate raises health concerns as a precursor to endogenous nitrosation,^57^ *N*‐nitrosamines are also detected directly in water leading to regulatory limits worldwide yet contribute only ∼3% of total exposure.^58^, ^59^ Possible confounding from other neurotoxic contaminants such as aluminium^60^ and arsenic^61^ cannot be excluded. Given these findings and the complexity of underlying mechanisms, further research and consideration of stricter water nitrate regulations are needed.

Source‐dependent associations with dementia were two‐ to four‐fold larger for early‐onset than total dementia. However, early‐onset cases were fewer (191 vs 4559), and diagnoses before the age of 50 could not be examined. Associations weakened with increasing age at diagnosis, consistent with a 12‐year prospective study of 250,000 to 300,000 individuals reporting stronger associations between sociodemographic and lifestyle factors and early‐onset versus late‐onset dementia, whereas apolipoprotein E ε4 allele carriage showed stronger associations with late‐onset cases.^25^ Although late‐onset dementia is more strongly associated with vascular damage and dysfunction,^62^ which depend on NO bioavailability, the stronger association between plant‐sourced nitrate and nitrite and early‐onset dementia may reflect greater vascular responsiveness earlier in life.^62^ These findings warrant further investigation, as an estimated four million people aged 30 to 64 years currently live with early‐onset dementia worldwide, with 370,000 new diagnoses annually.^63^

The finding that plant‐sourced nitrate was protective only in participants with lower vitamin C and polyphenol intake may appear to contradict the hypothesis that these antioxidants inhibit endogenous *N*‐nitrosamine formation.^6^ However, this association is biologically plausible when contextualized within the complexity of diet and neuroprotection. Plant nitrate is consumed alongside compounds such as flavonoids that independently support vascular health, reduce oxidative stress, and modulate neuroinflammation.^5^, ^48^ In individuals with higher vitamin C and polyphenol intake, any added benefit of nitrate may be attenuated by a ceiling effect, whereas in participants with lower vitamin C and polyphenol intake and likely poorer overall diet quality, plant nitrate may provide distinct vasoprotective benefits, improving endothelial function and cerebral perfusion.^64^, ^65^ The nitrosation‐inhibiting effects of vitamin C and polyphenols matter more for nitrate and nitrite consumption from animal sources that co‐occur with amines and heme iron‐promoting *N*‐nitrosamine formation.^17^ The observed association of increased dementia risk with additive‐permitted meat nitrate intake among higher polyphenol consumers should be interpreted cautiously, as these results may reflect chance findings given small subgroup sizes and multiple comparisons. As vitamin C and polyphenols likely have little influence on preformed *N*‐nitrosamines in processed meats,^17^ these findings underscore the complexity of dietary interactions and indicate that the “NO versus *N*‐nitrosamine” hypothesis is not a simple one but requires consideration of other influences such as source‐specific effect modification by promoters and inhibitors of nitrosation.

This study has notable strengths, including a large cohort, up to 27 years of follow‐up, and minimal attrition, allowing for a significant number of dementia cases to be recorded. Comprehensive food databases were used for nitrate and nitrite content, while integration of longitudinal, residence‐specific water nitrate measures provided robust assessment of water exposure. Because it was an observational study, causality cannot be inferred, and findings should be interpreted as hypothesis generating. Disentangling the effects of nitrate from the broader dietary matrices is challenging, as nitrate intake correlates with healthy lifestyle and favorable socioeconomic factors. Despite extensive adjustments, residual confounding by these and environmental exposures cannot be excluded. Dietary data were only captured at baseline, and changes in intake over 27 years may have introduced non‐differential misclassification, likely biasing estimates toward the null, consistent with stronger associations observed in 10‐year analyses. Nitrate and nitrite estimates based on a FFQ may have recall bias, and the contribution of less common high‐nitrate foods may have been missed, further limiting exposure precision. The relatively low water nitrate range may not reflect exposures across Denmark, where about 10% of the population is exposed to levels exceeding 9 mg/L.^66^ We also lacked data on drinking water nitrate levels at participants’ workplaces. Other than being excluded at baseline for a dementia diagnosis, there was no information on cognitive impairment at baseline, which may have impacted the ability to recall dietary habits accurately. Dementia case ascertainment based on clinical diagnosis or medication records may have missed cases, and subtypes could not be differentiated. Finally, as participants were predominantly Caucasian, generalizability to other populations is limited, underscoring the need for replication in more ethnically and geographically diverse cohorts.

In this Danish prospective cohort, we observed source‐dependent associations between dietary nitrate and nitrite intake and dementia risk. Moderate, habitual intake of nitrate‐ and nitrite‐rich plants was associated with lower incident dementia rates, whereas higher intake of animal‐based sources and drinking water nitrate, even below regulatory limits, was associated with higher risks. Associations were more pronounced for early‐onset dementia. Although these findings should be interpreted within the broader context of the complex dietary and lifestyle influences, they support the hypothesis that nitrate and nitrite health effects vary by source, extend health considerations beyond cancer, and highlight the need for further research to clarify associations and guide preventive strategies for dementia.

## CONFLICT OF INTEREST STATEMENT

The authors declare no conflicts of interest. Author disclosures are available in the supporting information.

## CONSENT STATEMENT

All human subjects provided informed consent.

## Supporting information



Supporting information

Supporting information

## Data Availability

Data described in the manuscript, code book, and analytic code will be made available upon request pending application and approval by the Diet, Cancer and Health Steering Committee at the Danish Cancer Society.
