# Supplementary material for: Source‐specific nitrate intake and incident dementia in the Danish Diet, Cancer and Health Study
Source: Alzheimers Dement. 2025 Dec 19;21(12):e70995. doi: 10.1002/alz.70995 (PMC12715690; doi:10.1002/alz.70995)
Supplement: Supplementary file 1 — Supporting information [file ALZ-21-e70995-s001.docx]

**Source-specific nitrate intake and incident dementia in the Danish Diet, Cancer, and Health Study**

*Bondonno et al.*

SUPPLEMENTARY MATERIAL

| **Supplementary Table 1.** Definitions for determining prevalent comorbidities at baseline | |
| --- | --- |
| **Prevalent disease** | **Definition** |
| Ischemic heart disease | ICD-8 diagnosis [410-414] or ICD-10 diagnosis [I20-I25] in the Danish National Patient Register prior to baseline |
| Ischemic stroke | ICD-8 diagnosis [433-434] or ICD-10 diagnosis [I63] in the Danish National Patient Register prior to baseline |
| Peripheral artery disease | ICD-8 diagnosis [440-444] or ICD-10 diagnosis [I70-I74] in the Danish National Patient Register prior to baseline |
| Heart failure | ICD-8 diagnosis [4270-4271] or ICD-10 diagnosis [I42, I50, I110, J81] in the Danish National Patient Register prior to baseline |
| Atrial fibrillation | ICD-8 diagnosis [42793-42794] or ICD-10 diagnosis [I48] in the Danish National Patient Register prior to baseline |
| Chronic obstructive pulmonary disease (COPD) | ICD-8 diagnosis [491-493] or ICD-10 diagnosis [J42-J44] in the Danish National Patient Register or a record of COPD in the Register for Selected Chronic Diseases (RUKS) registry^1^ prior to baseline |
| Diabetes | A record of either type 1 or type 2 diabetes in the Register for Selected Chronic Diseases (RUKS) registry^1^ prior to baseline |
| Chronic kidney disease (CKD) | ICD-8 diagnosis [580-584] or ICD-10 diagnosis [N02-N08, N11-N12, N14, N18-N19, N26, N158-N160, N162-N164, N168, Q61, E102, E112, E132, E142, I120, M321B] in the Danish National Patient Register or a record of CKD in the Register for Selected Chronic Diseases (RUKS) registry^1^ prior to baseline. |

ICD; International Classification of Diseases [the 8^th^ revision (ICD-8) until 1993 and the 10^th^ revision (ICD-10) from 1994 to present]. Only type A & B diagnoses recorded in the Danish National Patient Register were accepted as valid diagnostic records. ^1^ Algorithms described in more detail here (1).


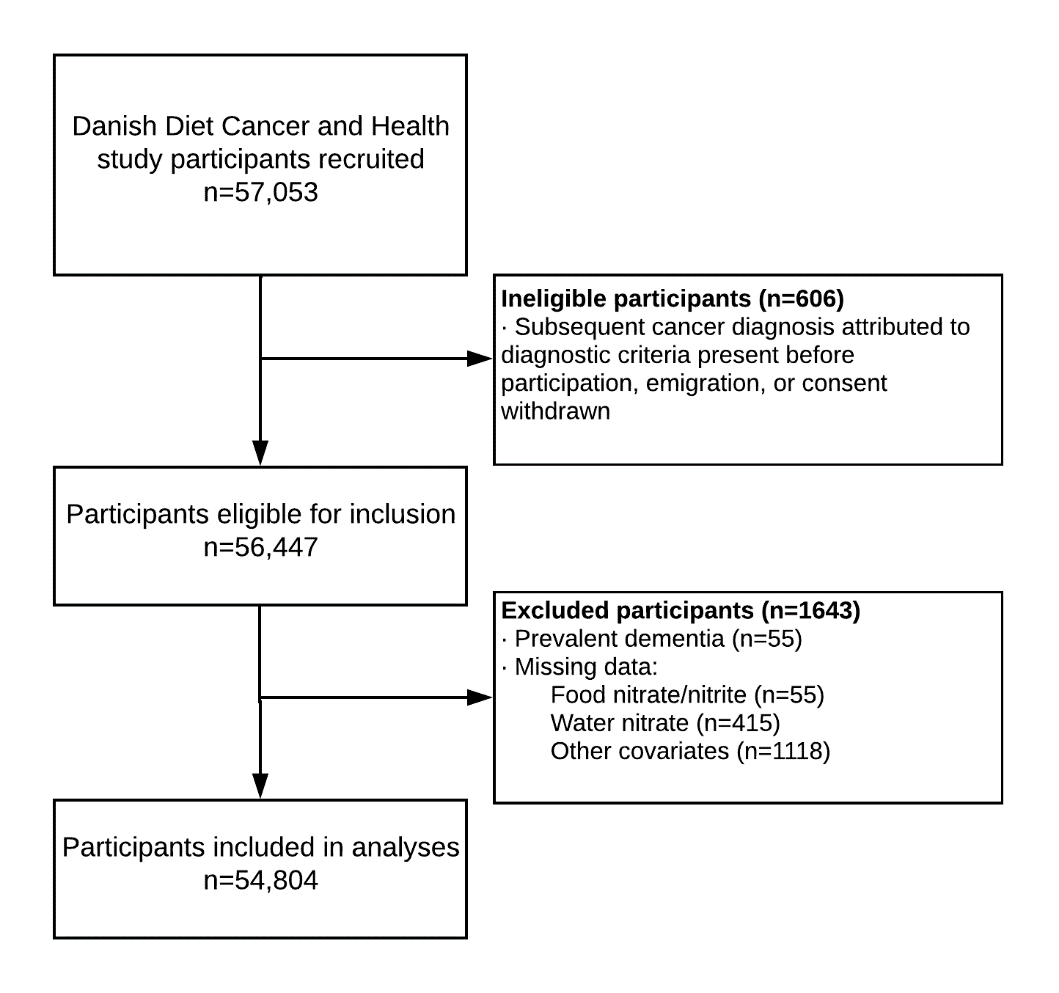


**Supplementary Figure 1**: CONSORT flow diagram of the Danish Diet Cancer and Health study participants included in present study


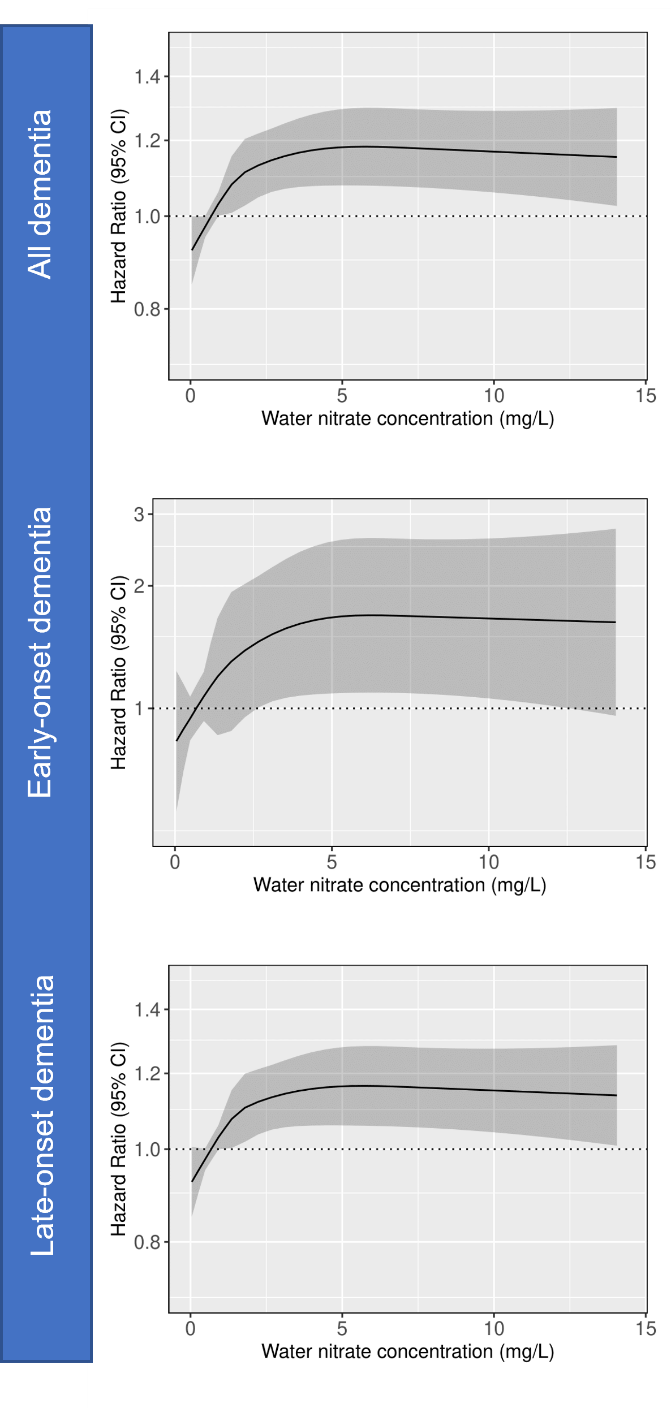


**Supplementary Figure 2.** Cubic spline curves depicting the association between water nitrate concentration at baseline and incident dementia in participants of the Danish Diet Cancer and Health cohort (n=54,804). Hazard ratios and 95% Confidence Intervals (CIs) are based on Cox proportional hazards models adjusted for age, sex, BMI, smoking status, smoking packyears, alcohol consumption, education level, physical activity level, living situation, and intakes of wholegrains, refined grains, red meat, processed meat, poultry, dairy, fish, vegetables, fruits, vegetable oils, sugar and confectionary, soft drinks, coffee, tea, and tap water intake (Model 3), and are comparing the specific level of the exposure (horizontal axis) to the median intake for participants in the lowest quintile.

| **Supplementary Table 2. Hazards ratio of dementia by quintiles of drinking water nitrate concentration from time-updated analyses** | | | | | |
| --- | --- | --- | --- | --- | --- |
|  | Q1 | Q2 | Q3 | Q4 | Q5 |
| Concentration* (mg/L) | 0.8 (0.7 – 1.0) | 1.4 (1.4 – 1.4) | 1.8 (1.8 – 2.0) | 2.4 (2.3 – 2.9) | 5.1 (4.2 – 7.1) |
| All dementia | |  |  |  |  |
| No. events | 424 | 572 | 526 | 561 | 547 |
| Model 1 | Ref. | 1.08 (1.00, 1.17) | 1.12 (1.02, 1.24) | 1.17 (1.06, 1.28) | 1.20 (1.06, 1.36) |
| Model 2 | Ref. | 1.08 (1.00, 1.17) | 1.12 (1.01, 1.23) | 1.15 (1.04, 1.26) | 1.16 (1.03, 1.32) |
| Model 3 | Ref. | 1.08 (0.99, 1.17) | 1.12 (1.01, 1.23) | 1.15 (1.04, 1.26) | 1.16 (1.03, 1.32) |
| Hazard ratios (95% Confidence Intervals; CIs) for all dementia, obtained from restricted cubic splines based on Cox proportional hazards models with age as the underlying timescale. Model 1 included sex and time since study entrance; Model 2 included sex, time since study entrance, BMI, smoking status, smoking packyears, alcohol consumption, education level, physical activity level, living situation and baseline tap water intake; Model 3 adjusted for the covariates in Model 2 plus intakes of wholegrains, refined grains, red meat, processed meat, poultry, dairy, fish, vegetables, fruits, vegetable oils, sugar and confectionary, soft drinks.  *Baseline 15-year average concentration of drinking water nitrate presented as median (Interquartile Range; IQR) | | | | | |


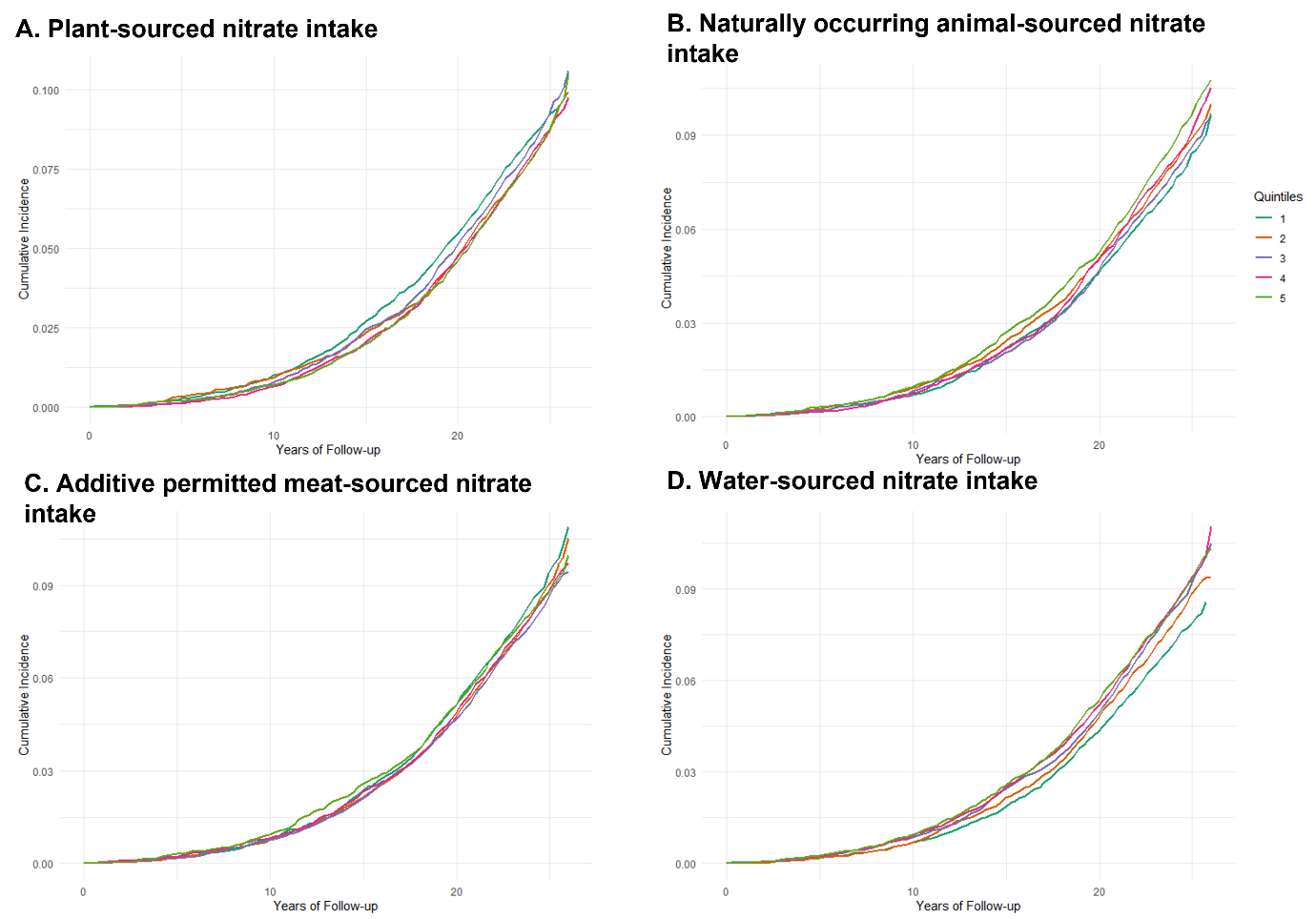


**Supplementary Figure 3.** Cumulative incidence of dementia accounting for death as a competing risk using the Aalen-Johansen estimator, across quintiles of **A.** plant-sourced nitrate, **B.** naturally occurring animal-sourced nitrate, **C.** additive-permitted meat-sourced nitrate and **D.** tap water-sourced nitrate intakes.

| **Supplementary Table 3. Hazards ratio of dementia by quintiles of source-dependent nitrate stratified by age** | | | | | |
| --- | --- | --- | --- | --- | --- |
|  | **Q1** | **Q2** | **Q3** | **Q4** | **Q5** |
|  | **n=10,961** | **n=10,961** | **n=10,960** | **n=10,961** | **n=10,961** |
| **Plant-sourced nitrate** | |  |  |  |  |
| < 65 years | Ref. | 0.87 (0.68, 1.12) | 0.83 (0.62, 1.13) | 0.81 (0.55, 1.20) | 0.69 (0.44, 1.08) |
| 65 ≥ and < 70 years | Ref. | 0.89 (0.74, 1.07) | 0.84 (0.68, 1.05) | 0.83 (0.64, 1.07) | 0.90 (0.68, 1.19) |
| 70 ≥ and < 75 years | Ref. | 0.88 (0.78, 1.00) | 0.86 (0.75, 1.00) | 0.88 (0.74, 1.05) | 0.83 (0.68, 1.01) |
| 75 ≥ and < 80 years | Ref. | 0.97 (0.88, 1.07) | 0.97 (0.86, 1.10) | 0.99 (0.86, 1.14) | 0.98 (0.84, 1.14) |
| ≥ 80 years | Ref. | 0.91 (0.82, 1.00) | 0.90 (0.80, 1.01) | 0.93 (0.81, 1.06) | 0.91 (0.78, 1.05) |
| **Naturally occurring animal-sourced nitrate** | | |  |  |  |
| < 65 years | Ref. | 1.28 (0.95, 1.73) | 1.52 (1.01, 2.27) | 1.66 (1.11, 2.47) | 1.73 (1.12, 2.66) |
| 65 ≥ and < 70 years | Ref. | 1.16 (0.96, 1.40) | 1.25 (0.97, 1.62) | 1.28 (0.99, 1.65) | 1.32 (1.00, 1.74) |
| 70 ≥ and < 75 years | Ref. | 1.02 (0.90, 1.16) | 1.04 (0.88, 1.22) | 1.05 (0.89, 1.24) | 1.06 (0.88, 1.27) |
| 75 ≥ and < 80 years | Ref. | 1.04 (0.94, 1.14) | 1.06 (0.93, 1.21) | 1.08 (0.95, 1.23) | 1.12 (0.97, 1.29) |
| ≥ 80 years | Ref. | 1.03 (0.94, 1.13) | 1.07 (0.96, 1.20) | 1.11 (0.98, 1.27) | 1.17 (1.00, 1.36) |
| **Additive permitted meat-sourced nitrate** | | |  |  |  |
| < 65 years | Ref. | 1.14 (0.84, 1.55) | 1.25 (0.82, 1.90) | 1.30 (0.86, 1.97) | 1.40 (0.87, 2.25) |
| 65 ≥ and < 70 years | Ref. | 0.93 (0.78, 1.11) | 0.87 (0.68, 1.11) | 0.83 (0.65, 1.06) | 0.81 (0.60, 1.10) |
| 70 ≥ and < 75 years | Ref. | 0.99 (0.87, 1.12) | 1.04 (0.88, 1.23) | 1.16 (0.97, 1.37) | 1.27 (1.04, 1.56) |
| 75 ≥ and < 80 years | Ref. | 1.05 (0.95, 1.16) | 1.09 (0.96, 1.25) | 1.13 (0.99, 1.30) | 1.17 (1.00, 1.38) |
| ≥ 80 years | Ref. | 0.99 (0.90, 1.09) | 1.00 (0.88, 1.13) | 1.02 (0.90, 1.16) | 1.01 (0.87, 1.18) |
| **Tap water-sourced nitrate** | | |  |  |  |
| < 65 years | Ref. | 1.20 (0.91, 1.59) | 1.39 (0.90, 2.14) | 1.45 (0.98, 2.13) | 1.53 (0.99, 2.37) |
| 65 ≥ and < 70 years | Ref. | 1.06 (0.88, 1.28) | 1.14 (0.85, 1.52) | 1.22 (0.94, 1.58) | 1.31 (0.98, 1.75) |
| 70 ≥ and < 75 years | Ref. | 1.08 (0.95, 1.22) | 1.15 (0.95, 1.41) | 1.20 (1.01, 1.44) | 1.26 (1.03, 1.54) |
| 75 ≥ and < 80 years | Ref. | 0.99 (0.90, 1.10) | 1.01 (0.86, 1.18) | 1.05 (0.91, 1.21) | 1.11 (0.95, 1.30) |
| ≥ 80 years | Ref. | 0.97 (0.88, 1.08) | 0.97 (0.83, 1.13) | 1.00 (0.87, 1.15) | 1.03 (0.88, 1.20) |
| Hazard ratios (HRs) and 95% Confidence Intervals (CIs) for dementia during up to 27 years of follow up, obtained from Cox proportional hazards models. HRs for each quintile (30th, 50th, 70th, and 90th percentiles) were derived from restricted cubic spline models, with the 10^th^ percentile as the reference. HRs presented are for Model 3 which included age, sex, BMI, smoking status, smoking packyears, alcohol consumption, education level, physical activity level and living situation; plus intakes of a) red meat, processed meat, poultry, dairy, fish, sugar and confectionary, soft drinks, refined grains, coffee, and tea when plant-sourced nitrate or nitrite were the exposures of interest, b) wholegrains, refined grains, vegetables, fruits, vegetable oils, sugar and confectionary, soft drinks, refined grains, coffee, and tea when animal-sourced nitrate or nitrite were the exposures of interest, and c) wholegrains, refined grains, red meat, processed meat, poultry, dairy, fish, vegetables, fruits, vegetable oils, sugar and confectionary, and soft drinks when water-sourced nitrate was the exposure of interest.  Exposure intakes are presented as median [Interquartile Range; IQR].  Participants: < 65 years: n = 191; 65 ≥ and < 70 years: n=436; 70 ≥ and < 75 years: n=961; 75 ≥ and < 80 years: n=1522; and ≥ 80 years: n=1640. | | | | | |

| **Supplementary Table 4. Hazards ratio of dementia by quintiles of source-dependent nitrate and nitrite intakes with follow-up period restricted to 10 years** | | | | | |
| --- | --- | --- | --- | --- | --- |
|  | **Q1** | **Q2** | **Q3** | **Q4** | **Q5** |
|  | **n=10,961** | **n=10,961** | **n=10,960** | **n=10,961** | **n=10,961** |
| **Plant-sourced nitrate** | |  |  |  |  |
| Intake (mg/d) | 22 [18, 25] | 34 [31, 36] | 44 [41, 47] | 56 [53, 60] | 77 [70, 88] |
| No. events | 111 | 103 | 88 | 71 | 77 |
| Model 1 | Ref. | 0.86 (0.73, 1.02) | 0.77 (0.64, 0.93) | 0.70 (0.56, 0.88) | 0.62 (0.48, 0.80) |
| Model 2 | Ref. | 0.94 (0.79, 1.11) | 0.89 (0.73, 1.09) | 0.84 (0.66, 1.06) | 0.75 (0.58, 0.98) |
| Model 3 | Ref. | 0.95 (0.80, 1.13) | 0.88 (0.71, 1.08) | 0.78 (0.6, 1.01) | 0.65 (0.49, 0.87) |
| **Vegetable-sourced nitrate** | | |  |  |  |
| Intake (mg/d) | 17 [13, 19] | 26 [24, 29] | 35 [33, 38] | 46 [43, 49] | 64 [58, 74] |
| No. events | 112 | 110 | 88 | 71 | 69 |
| Model 1 | Ref. | 0.80 (0.68, 0.94) | 0.70 (0.58, 0.85) | 0.64 (0.51, 0.81) | 0.56 (0.43, 0.72) |
| Model 2 | Ref. | 0.86 (0.73, 1.01) | 0.79 (0.65, 0.96) | 0.75 (0.59, 0.95) | 0.65 (0.50, 0.85) |
| Model 3 | Ref. | 0.87 (0.73, 1.03) | 0.78 (0.63, 0.96) | 0.70 (0.54, 0.90) | 0.57 (0.43, 0.76) |
| **Plant-sourced nitrite** | | |  |  |  |
| Intake (mg/d) | 0.4 [0.3, 0.5] | 0.6 [0.6, 0.7] | 0.8 [0.7, 0.9] | 1.0 [1.0, 1.1] | 1.5 [1.3, 1.7] |
| No. events | 113 | 100 | 76 | 77 | 84 |
| Model 1 | Ref. | 0.84 (0.70, 0.99) | 0.73 (0.60, 0.89) | 0.67 (0.54, 0.83) | 0.67 (0.52, 0.86) |
| Model 2 | Ref. | 0.93 (0.78, 1.11) | 0.87 (0.71, 1.07) | 0.82 (0.65, 1.02) | 0.83 (0.64, 1.07) |
| Model 3 | Ref. | 0.96 (0.80, 1.15) | 0.88 (0.70, 1.09) | 0.78 (0.61, 1.00) | 0.76 (0.57, 1.00) |
| **Vegetable-sourced nitrite** | | |  |  |  |
| Intake (mg/d) | 0.2 [0.2, 0.2] | 0.3 [0.3, 0.4] | 0.5 [0.4, 0.5] | 0.6 [0.6, 0.6] | 0.8 [0.8, 1.0] |
| No. events | 120 | 97 | 86 | 82 | 65 |
| Model 1 | Ref. | 0.83 (0.7, 0.98) | 0.73 (0.60, 0.89) | 0.68 (0.54, 0.85) | 0.56 (0.44, 0.73) |
| Model 2 | Ref. | 0.89 (0.75, 1.06) | 0.84 (0.68, 1.03) | 0.79 (0.62, 1.01) | 0.66 (0.51, 0.87) |
| Model 3 | Ref. | 0.92 (0.77, 1.10) | 0.84 (0.68, 1.05) | 0.76 (0.58, 0.98) | 0.59 (0.44, 0.80) |
| **Naturally occurring animal-sourced nitrate** | | |  |  |  |
| Intake (mg/d) | 2.7 [2.3, 3.1] | 4.0 [3.7, 4.3] | 5.4 [5.0, 5.9] | 7.5 [6.9, 8.1] | 10.7 [9.5, 13.5] |
| No. events | 77 | 100 | 87 | 81 | 105 |
| Model 1 | Ref. | 1.07 (0.89, 1.28) | 1.10 (0.86, 1.4) | 1.09 (0.86, 1.39) | 1.11 (0.86, 1.44) |
| Model 2 | Ref. | 1.11 (0.93, 1.34) | 1.19 (0.93, 1.51) | 1.22 (0.96, 1.54) | 1.25 (0.96, 1.62) |
| Model 3 | Ref. | 1.23 (1.02, 1.48) | 1.36 (1.06, 1.75) | 1.36 (1.06, 1.75) | 1.37 (1.04, 1.80) |
| **Additive permitted meat-sourced nitrate** | | | |  |  |
| Intake (mg/d) | 0.1 [0.1, 0.1] | 0.2 [0.2, 0.2] | 0.3 [0.3, 0.3] | 0.4 [0.4, 0.5] | 0.7 [0.6, 0.9] |
| No. events | 89 | 86 | 83 | 90 | 102 |
| Model 1 | Ref. | 0.90 (0.76, 1.07) | 0.87 (0.69, 1.10) | 0.90 (0.71, 1.13) | 0.97 (0.74, 1.28) |
| Model 2 | Ref. | 0.91 (0.76, 1.08) | 0.86 (0.68, 1.09) | 0.85 (0.67, 1.08) | 0.87 (0.66, 1.16) |
| Model 3 | Ref. | 0.98 (0.82, 1.17) | 0.96 (0.75, 1.23) | 0.95 (0.74, 1.22) | 0.97 (0.72, 1.31) |
| **Naturally occurring animal-sourced nitrite** | | | |  |  |
| Intake (mg/d) | 0.3 [0.3, 0.4] | 0.5 [0.4, 0.5] | 0.6 [0.5, 0.6] | 0.7 [0.6,0.7] | 0.9 [0.8, 1.1] |
| No. events | 74 | 90 | 96 | 88 | 102 |
| Model 1 | Ref. | 1.13 (0.94, 1.36) | 1.13 (0.91, 1.41) | 1.09 (0.85, 1.39) | 1.12 (0.84, 1.47) |
| Model 2 | Ref. | 1.16 (0.96, 1.40) | 1.17 (0.93, 1.46) | 1.11 (0.87, 1.42) | 1.11 (0.83, 1.47) |
| Model 3 | Ref. | 1.29 (1.06, 1.56) | 1.36 (1.07, 1.72) | 1.31 (1.01, 1.71) | 1.33 (0.97, 1.81) |
| **Additive permitted meat-sourced nitrite** | | |  |  |  |
| Intake (mg/d) | 0.0 [0.0, 0.0] | 0.0 [0.0, 0.1] | 0.1 [0.1, 0.1] | 0.1 [0.1, 0.1] | 0.2 [0.2, 0.2] |
| No. events | 83 | 83 | 99 | 85 | 100 |
| Model 1 | Ref. | 0.99 (0.83, 1.18) | 0.97 (0.77, 1.24) | 0.97 (0.76, 1.22) | 0.99 (0.75, 1.30) |
| Model 2 | Ref. | 0.99 (0.83, 1.18) | 0.96 (0.75, 1.22) | 0.91 (0.71, 1.15) | 0.88 (0.66, 1.17) |
| Model 3 | Ref. | 1.07 (0.89, 1.28) | 1.07 (0.84, 1.37) | 1.01 (0.79, 1.30) | 0.97 (0.72, 1.31) |
| **Tap water-sourced nitrate** | | |  |  |  |
| Intake (mg/d) | 0 [0, 0.1] | 0.3 [0.2, 0.4] | 0.8 [0.6, 0.9] | 1.5 [1.1, 1.8] | 3.3 [2.6, 4.9] |
| No. events | 82 | 87 | 93 | 91 | 97 |
| Model 1 | Ref. | 1.04 (0.86, 1.25) | 1.09 (0.82, 1.45) | 1.17 (0.90, 1.51) | 1.26 (0.95, 1.68) |
| Model 2 | Ref. | 1.12 (0.93, 1.34) | 1.22 (0.92, 1.63) | 1.27 (0.98, 1.65) | 1.33 (1.00, 1.77) |
| Model 3 | Ref. | 1.11 (0.92, 1.34) | 1.21 (0.91, 1.62) | 1.26 (0.97, 1.63) | 1.31 (0.98, 1.75) |
| Hazard ratios (HRs) and 95% Confidence Intervals (Cis) for total dementia during up to 10 years of follow up, obtained from Cox proportional hazards models. HRs and 95% CIs for each quintile (30th, 50th, 70th, and 90th percentiles) were derived from restricted cubic spline models, with the 10^th^ percentile as the reference. Model 1 included age and sex; Model 2 included age, sex, BMI, smoking status, smoking packyears, alcohol consumption, education level, physical activity level and living situation; Model 3 adjusted for the covariates in Model 2 plus intakes of a) red meat, processed meat, poultry, dairy, fish, sugar and confectionary, soft drinks, refined grains, coffee, and tea when plant-sourced nitrate or nitrite were the exposures of interest, b) wholegrains, refined grains, vegetables, fruits, vegetable oils, sugar and confectionary, soft drinks, refined grains, coffee, and tea when animal-sourced nitrate or nitrite were the exposures of interest and c) wholegrains, refined grains, red meat, processed meat, poultry, dairy, fish, vegetables, fruits, vegetable oils, sugar and confectionary, and soft drinks when water-sourced nitrate was the exposure of interest.  Exposure intakes are presented as median [Interquartile Range; IQR]. | | | | | |

**References**

1. Sundhedsdatastyrelsen [Danish Board of Health Data], Algoritmer for udvalgte kroniske sygdomme og svære psykiske lidelser [Algorithms for selected chronic diseases and severe psychiatric conditions]. <https://www.esundhed.dk/Dokumentation/-/media/Files/Publikationer/Emner/Operationer-og-diagnoser/Udvalgte-kroniske-sygdomme-svaere-psykiske-lidelser/RUKS-Algoritmer-2021.ashx?la=da&hash=34DEC5295DF1442A92AD711C43EDD7C2> Accessed 21 Apr 2023.
